# Effects of influential points and sample size on the selection and replicability of multivariable fractional polynomial models

**DOI:** 10.1186/s41512-023-00145-1

**Published:** 2023-04-18

**Authors:** Willi Sauerbrei, Edwin Kipruto, James Balmford

**Affiliations:** Institute of Medical Biometry and Statistics, Faculty of Medicine and Medical Center, University of Freiburg, Freiburg, Germany

**Keywords:** Continuous variable, Fractional polynomial, Influential point, Model building, Sample size, Simulated data

## Abstract

**Background:**

The multivariable fractional polynomial (MFP) approach combines variable selection using backward elimination with a function selection procedure (FSP) for fractional polynomial (FP) functions. It is a relatively simple approach which can be easily understood without advanced training in statistical modeling. For continuous variables, a closed test procedure is used to decide between no effect, linear, FP1, or FP2 functions. Influential points (IPs) and small sample sizes can both have a strong impact on a selected function and MFP model.

**Methods:**

We used simulated data with six continuous and four categorical predictors to illustrate approaches which can help to identify IPs with an influence on function selection and the MFP model. Approaches use leave-one or two-out and two related techniques for a multivariable assessment. In eight subsamples, we also investigated the effects of sample size and model replicability, the latter by using three non-overlapping subsamples with the same sample size. For better illustration, a structured profile was used to provide an overview of all analyses conducted.

**Results:**

The results showed that one or more IPs can drive the functions and models selected. In addition, with a small sample size, MFP was not able to detect some non-linear functions and the selected model differed substantially from the true underlying model. However, when the sample size was relatively large and regression diagnostics were carefully conducted, MFP selected functions or models that were similar to the underlying true model.

**Conclusions:**

For smaller sample size, IPs and low power are important reasons that the MFP approach may not be able to identify underlying functional relationships for continuous variables and selected models might differ substantially from the true model. However, for larger sample sizes, a carefully conducted MFP analysis is often a suitable way to select a multivariable regression model which includes continuous variables. In such a case, MFP can be the preferred approach to derive a multivariable descriptive model.

**Supplementary Information:**

The online version contains supplementary material available at 10.1186/s41512-023-00145-1.

## Introduction

In modeling observational data aimed at identifying predictors of an outcome and gaining insight into the relationship between the predictors and the outcome, the process of building a model for description consists of two components: variable selection to identify the subset of “important” predictors, and identification of possible non-linearity in continuous predictors. The ultimate aim is to build a model which is satisfactory in terms of model fit, interpretable from the subject matter point of view, robust to minor variations in the current data, predictive in new data, and parsimonious [1].

In model building, many researchers typically assume a linear function for continuous variables (perhaps after applying a “standard” transformation such as log) or divide the variable into several categories. If the assumption of linearity is incorrect, it may prevent the detection of a stronger effect or even cause the effects to be mismodeled. Categorization of continuous variables, which has the effect of modeling (implausible) step functions, is common but widely criticized [1–4] and will not be considered further.

Fractional polynomials have been proposed as a simple method of dealing with non-linearity [1, 5–7]. First-degree (FP1, single power) functions are monotonic, whereas second-degree (FP2, two powers) functions can represent a variety of curve shapes with a single maximum or minimum. Models with degree higher than two are rarely required in practice. Fractional polynomials can be viewed as a compromise between conventional polynomials (e.g., quadratic functions) and non-linear curves generated by flexible modeling techniques such as spline functions, but without the inflexibility of the former or the potential instability of the latter. FPs are global functions that cannot handle local features, unlike several “flavors” of splines, e.g., restricted regression splines [8], penalized regression splines [9], smoothing splines [10], and p-splines [11]. Being global functions makes FPs more stable than local-influence models, which have a higher capacity for model fit but lower transferability and relative instability [12, 13].

The multivariable fractional polynomial (MFP) approach combines backward elimination with a three-step closed test procedure (the function selection procedure, or FSP) to select the most appropriate functional form for continuous variables from the proposed class of fractional polynomial functions (8 FP1 and 36 FP2). In this paper, several issues that may affect the identification and estimation of non-linear functions as well as model replicability were considered. The presence of covariate outliers, or IPs, may have an undue influence on the chosen model. In MFP, IPs are single or pairs (triples) of observations which have an unduly large influence on the selection of an FP function for a particular variable or the selected model [14]. Diagnostic plots were used to show how to identify IPs. We are not aware of any paper discussing the role of IPs in the selection of variables and functional forms for continuous variables.

In addition to the approach in the book by Royston and Sauerbrei [1], we discussed an extension to considering pairs of IPs and proposed two approaches for identifying IPs in multivariable models. We concentrated on the identification of IPs and illustrated their effects on functions and models selected by comparing results for data with and without IPs. IPs were eliminated and potential ways (e.g., truncation or preliminary transformation) to handle IPs in real data were not discussed. In real-world data, handling of IPs depends strongly on the specific study and main aim of a model. We also considered model replicability across datasets. This is an important aspect of multivariable modeling, particularly in the context of IPs, where the presence of an extreme value of a single covariate may affect the functions selected for that variable, correlated variables, and the overall model. Finally, the effect of sample size was investigated since the selection of variables and functions within the MFP procedure uses test statistics which depend strongly on the sample size. In small samples, variables with moderate or weak effects may be incorrectly eliminated or linear functions may be chosen instead of more realistic non-linear functions.

To assess whether MFP selects the “true” underlying model or a model which is close to it, it is imperative to use simulated data in which the parameters are known. In this paper, we used data from the ART study (ART denoting “artificial”, [1], Chap.10) which consisted of 5000 simulated observations. A subset of the ART data (*n*=250) were used as the “main” dataset to illustrate on how to work with MFP, including sections on model criticism. We conducted investigations in additional subsets (3 datasets, each of 250 observations) to examine function replicability and the influence of sample size (3 datasets of 125, 250 and 500 observations, respectively) but only selected parts are shown, see “Data not shown” in Additional file 1: Table A1. Based on the key principles of plasmode data sets [15], the distribution of the predictors in the ART study and their correlation structure was informed by a real study from the German Breast Cancer Study Group (GBSG), as described in a number of earlier publications [1, 6]. For more background on the GBSG study, the original data and data of the ART study is available at http://portal.uni-freiburg.de/imbi/Royston-Sauerbrei-book/index.html#datasets.

To improve the quality of reporting and provide a suitable overview of all analyses conducted, we extended the recently proposed ADEMP structure for simulation studies [16] with a structured display of analysis strategies and presentations, named MethProf-simu profile (see Table A1 in Additional file 1).

The rest of the paper is organized as follows. The section “The multivariable fractional polynomial procedure” introduces the MFP approach, while the section “Influential points and model replicability” discusses various aspects of investigations for IPs, model replicability, and sample size. The section “Design of the simulated data” introduces the simulated data. The results of several investigations for these data are presented in the section “Results”, followed by a discussion and conclusions. Several papers and a book have been published about MFP modeling. Therefore, we provide only a short explanation in the main text and give more details in the additional file (see section A1 in Additional file 1), intended for readers who are unfamiliar with the approach. Due to space limitations, many analyses and a case study have been relegated to the additional File (see Additional file 1).

## The multivariable fractional polynomial procedure

MFP is a multivariable model building approach which retains continuous predictors as continuous, finds non-linear functions if they are sufficiently supported by the data, and eliminates predictors with weak or no effects by backward elimination (BE) [1]. The two key components are variable selection with backward elimination and the function selection procedure (FSP) which selects an FP function for each continuous variable. The analyst must decide on a nominal significance level (α) for both components. The choice of these two significance levels has a strong influence on the complexity and stability of the final model [1, 17]. The same *α* level can be used for the two components, though it can differ. This decision strongly depends on the aim of the analysis. In MFP terminology, MFP(0.05) means an MFP model with both variables and functions selected at the 0.05 significance level while MFP(0.05, 0.01) means that variables are selected at the 0.05 level and functions at 0.01 level. In this paper, *α* = 0.05 was used for both components, but we also showed the threshold values for *α* = 0.01 and in some cases we discussed the result for this significance level in order to illustrate the importance of the chosen significance level on the identification of IPs and on the final model chosen. In principle, the MFP approach prefers simpler models because they transfer better to other settings and are more suited for practical use. This contrasts with local regression modeling (e.g., splines, kernel smoothers) which often starts and ends with more complex models [7].

The class of fractional polynomial (FP) functions is an extension of power transformations of a variable. For most applications, FP1 and FP2 functions are sufficient, and in this paper, we allowed FP2 to be the most complex function. For more details, see [1, 5] and the MFP website http://mfp.imbi.uni-freiburg.de/.

**Fig. 1:**
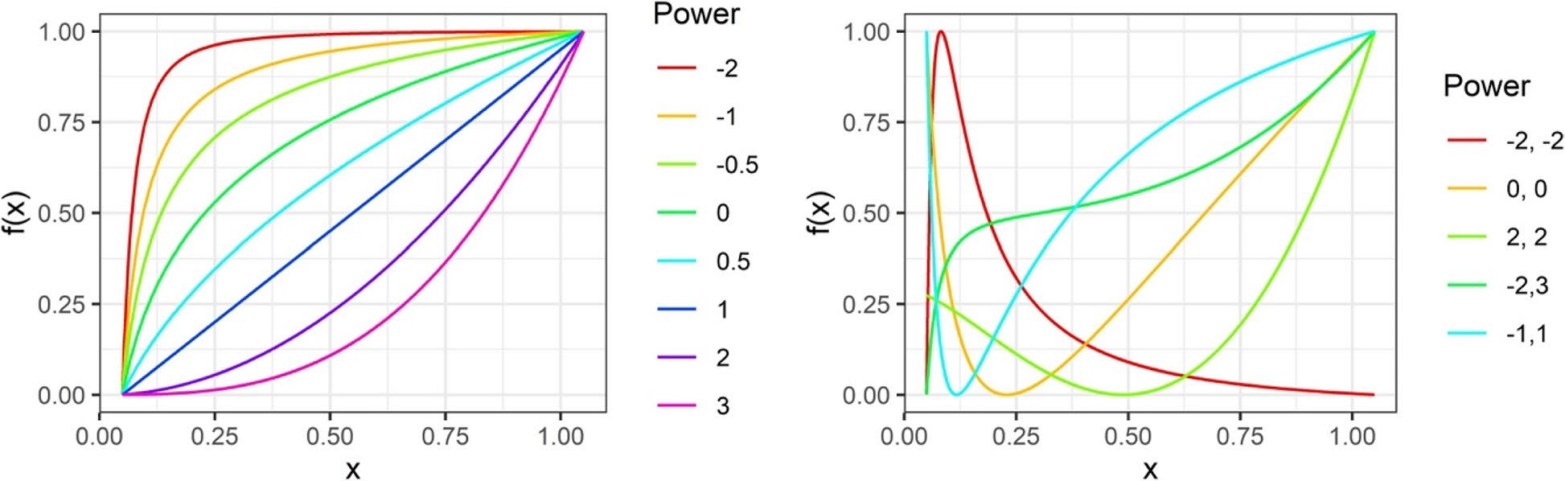
Schematic diagram of eight FP1 (left panel) and a subset of the 36 FP2 (right panel) functions

Fractional polynomial functions are defined in the following way:

$$FP1:\beta {x}^{p1}$$

$$FP2:{\beta}_1{x}^{p1}+{\beta}_2{x}^{p2},$$

with exponents *p*1 and *p*2 derived from a set *s* = {−2, −1, −0.5, 0, 0.5, 1, 2, 3}, where 0 stands for natural logarithm of *x*. If *p*1 = *p*2 (repeated powers), the *FP*2 function is defined as *β*_1_*x*^*p*^ + *β*_2_*x*^*p*^ log(*x*). Overall, the set of powers permits 44 models of which 8 are FP1 and 36 are FP2. The FP2 with powers (*p*1 = 1, *p*2 = 2) is equivalent to the quadratic function. While the permitted class of FP functions appears small, it includes very different types of shapes as illustrated in Fig. 1 for the eight FP1 powers and a subset of FP2 powers [1, 5].

In the MFP context, the FSP is conducted in a model adjusting for other variables (with their corresponding selected FP functions) currently in the model. The deviance (minus twice the maximized log likelihood) of the null model, the linear model, the best FP1 model, and the best FP2 model are compared if FP2 is the most complex function allowed. The extension to FP3 is straightforward but not considered here.

The procedure starts with a comparison of the best FP2 model with the null model (step 1). If significant, the procedure compares the best FP2 function with the linear model (step 2), and again if significant, the best-fitting FP1 is compared with the best FP2 (step 3). As interpretability, transportability, and practical usefulness are important components of MFP models, a non-linear FP function is chosen only if it fits the data significantly better than the linear function [7]. If non-linearity is required, a simpler (FP1) function is preferred to a more complex (FP2) function. The use of a closed test procedure ensures that the overall type 1 error rate of FSP is close to the nominal significance level [1, 18]. For MFP, it is important to note that if *α* = 1 for variable selection, then *x* is “forced” into the model and step 1 is redundant. If the best-fitting FP1 function is linear, step 3 is not required. For more details on FSP (see section A1 of the Additional file 1).

## Influential points and model replicability

The leverage of IPs may be high; for example, an FP2 model may be made statistically significant compared with FP1 by a single extreme observation of *x*. This is overfitting and should be avoided because inferences from a model strongly influenced by a single observation are unlikely to be reliable or generalize well to new data. After selecting a function using the FSP, it is important to check whether eliminating any individual observations (or pairs of observations) influences the significance of any of the three FSP tests and thus the selected function.

### Identification of influential points in univariable analysis

#### Diagnostic plot for single points

In accordance with the leave-one-out approach as proposed in the seminal article by Cook [19] on IPs, Royston and Sauerbrei [1] suggested that diagnostic plots be used to identify observations potentially influencing the selection of a function. Successively deleting each single observation from the original dataset, the deviance of the null model, the linear model, and the best-fitting FP1 and FP2 models was stored, and the deviance differences between model pairs were calculated (FP2 vs. null, FP2 vs. linear, and FP2 vs. FP1) and plotted against the deleted observation number or observed variable value. The ${\chi}_k^2$ critical values with *k* degrees of freedom and a significance level of *α* = 0.05, i.e., FP2 vs. null (9.488 for *k* = 4), FP2 vs. linear (7.815 for *k* = 3), and FP2 vs. FP1 (5.991 for *k* = 2) were used to decide whether a point was influential or not. For illustration, we also showed corresponding lines for significance level of *α*=0.01 with critical values of 13.277 (*k* = 4), 11.345 (*k* = 3), and 9.210 (*k* = 2). Observations which influence the choice of an FP model can be easily observed because their deletion changes the deviance difference, sometimes dramatically compared to the other observations. If the deviance difference is less than the ${\chi}_k^2$ threshold, there is evidence that the choice of the more complex model depends on this observation or observations and that a simpler model may be preferred. Since the threshold depends on *α*, an observation may be influential at the 0.05 level but not at the 0.01 level.

#### Diagnostic plot for combinations of two or more points

Royston and Sauerbrei [1] only discussed the identification of single IPs. The inclusion or exclusion of predictor variables in the model, as well as the functional forms selected can be influenced by the effect of particular combinations of two or more observations, which can lead to discrepant results. To extend the use of diagnostic plots to detect two or more IPs, the method described in subsection “Diagnostic plot for single points” was extended by successively deleting a subset of *d* observations from the original data, which led to *n* ! /(*d* ! (*n* − *d*)!) samples. To better understand the effects of IPs, we considered *d* = 2 because higher values can be computationally intensive due to a high number of possible combinations. For each pair (*i*, *j*) where *i* ≠ *j*, we constructed samples by removing the *i*th and *j*th data points from the original data and fitting fractional polynomial models. Box plots were used to summarize the deviance differences between model pairs for each combination. In total, we had 31,125 replicates generated from a sample size of 250 observations. Pairs containing one specific observation and a subset of the remaining observations are often on opposite sides of the threshold. Boxplots for subgroups of the 31,125 pairs can be used to illustrate the effect of influential pairs. As before, the ${\chi}_k^2$ threshold was used to determine whether pairs of observations were influential. The approach for searching for triples is straightforward and was not explored here. Obviously, it is computer intensive for larger sample sizes.

### Identification of influential points in multivariable analysis

Conducting diagnostic analyses for IPs in the multivariable modeling raises additional issues, and we illustrated two approaches. First, we checked for IPs in each covariate using the approach discussed in subsection “Diagnostic plot for single points”. Then all observations that were influential for at least one variable were eliminated, and the final MFP model was estimated using the reduced dataset. The second approach started with an MFP analysis of the full data set, followed by a check for IPs in the selected model. In principle, we exchanged the order of checking for IPs and deriving the MFP model. We did not check for IPs in variables excluded from the MFP model.

#### Univariable analyses to identify IPs followed by MFP on reduced data

Observations identified as IPs for at least one covariate in univariable models were eliminated and an MFP approach was used in data without IPs (the reduced dataset), with the results referred to as IPXu (IP in data X, univariable); in the next subsection, we used IPXm for a multivariable approach to avoid confusion. Although this process of identification uses the univariable analysis of each variable, the observations identified are also likely to influence a joint analysis of the variables. The effects of the observations identified as possible IPs were evaluated by comparing the estimated functions of multivariable models selected on the full data and reduced data.

#### MFP analysis followed by check for IPs

If the underlying model is multivariable, the IPs identified by univariable analysis may differ from those identified by multivariable analysis. Thus, another approach is to perform diagnostic analyses on the MFP model selected using all the data. While adjusting for all other variables in the selected model, the three tests of the FSP for each continuous variable were performed after successively deleting the *i*th observation (or a pair) as previously conducted in univariable analysis. The FP powers and parameter estimates from the selected MFP model were kept for the adjustment model, whereas Royston and Sauerbrei [1] kept the power terms but re-estimated regression coefficients in the reduced data. We used the notation IPXm to denote the MFP model in data X after the removal of IPs identified in the multivariable approach.

### Model replicability

A related issue to IPs is model stability and replicability. In this context, replicability means that the results of fitting MFP models to datasets generated from the same distribution should be identical or nearly identical in terms of variables and functions selected. We demonstrated the replicability of models by selecting MFP models in the three datasets (*n* = 250) sampled from the ART data: A250 (obs. 1–250), B250 (obs. 2001–2250), and C250 (obs. 3001–3250). As IPs have an impact on the selection of variables and functional forms, we compared the functions estimated from the data with and without IPs.

A single model is produced after a model selection procedure is applied to a set of candidate covariates. A very low *p*-value indicates that a covariate may have a stronger effect and is thus “stable,” in the sense that it has a high chance of being selected in similar datasets. For less significant covariates, selection may be more of a matter of chance and the model chosen may be influenced by the characteristics of a small number of observations. If the data is slightly altered, a different model may be selected. Studies assessing the stability of variable selection procedures using bootstrap resampling show that the variables with stronger effects are selected in the vast majority of bootstrap replications, whereas those with weak or “borderline significant” effects may enter the model at random [20, 21], and their inclusion can be heavily influenced by IPs.

### Influence of sample size

The MFP relies on significance tests for variable and function selection and the detection of non-linear functions requires a large sample size. The smaller the sample size (or in survival analysis, the fewer the number of events), the less likely a test is significant at any given significance level. In FSP, a linear function is the default, and if the sample size is insufficient, a variable may be eliminated or a linear function selected, even if the true function is very different. In the context of variable selection, a range of 10 to 25 observations per variable has been recommended in order to derive suitable models for description [8, 22]. Larger sample sizes are usually required for function selection to have sufficient power to reject a linear function in favor of a non-linear function.

When a non-linear function is required, Type II errors (falsely inferring a linear function; second test of FSP not significant) or even eliminating a variable (first test of FSP not significant) can be a serious problem in smaller samples. The effect of sample size on a model selected was demonstrated using different-sized subsets of the ART data, i.e., A125 (obs. 1–125), A250 (obs. 1–250), and A500 (obs. 1–500). For relatively large sample sizes (*n* = 500, about 41 observations per variable), model replicability was investigated by comparing the selected MFP models for datasets A500, B500 (obs. 2001–2500), and C500 (obs. 3001–3500). Based on a reviewers’ suggestion, we investigated the effects of IPs in a relatively large dataset (*n* = 1000; data D1000 (obs. 3501–4500)). In all data, checks for IPs were conducted and results compared after exclusion of IPs.

## Design of the simulated data

This section introduces the simulated data set used to illustrate the MFP approach and investigate the issues of IPs, model replicability, and sample size. The data are publicly available from the MFP website https://mfp.imbi.uni-freiburg.de/.

In the spirit of plasmode simulations [15], the ART data set is composed of 5000 simulated observations that mimic the GBSG breast cancer study in terms of the distribution of predictors and correlation structure (see Appendix A.2.2 in [1]). It has a continuous response variable *y*, and 10 covariates. The covariates include six continuous variables (x1, x3, x5, x6, x7, and x10), two binary variables (x2 and x8), and two 3-level categorical variables (x4 and x9), of which x4 is ordinal and x9 is nominal. For each of x4 and x9, two dummy variables with an ordinal (x4) and a categorical (x9) coding were used. The true model used to generate the ART data was given by

$$\begin{aligned} y&=-4+3.5{x}_1^{0.5}-0.25{x}_1-0.018{x}_3-0.4{x}_{4a}+4{x}_5^{-0.2}\\ &\quad+0.25\log \left({x}_6+1\right)+0.4{x}_8+0.021{x}_{10}+\epsilon \end{aligned}$$

where *ϵ* is the random noise assumed to be independent and identically distributed *N*(0, *σ*^2^) with *σ*^2^ = 0.49,resulting in *R*^*2*^ of about 0.50. There are five continuous variables and two categorical variables with an effect on the outcome. The power for variable x5 (−0.2) is not an element of a set of FP functions, and so can only be modeled approximately using the FP approach while a value of 1 was added to variable x6 before logarithm transformation due to 0 values. The contribution of each variable to the model fit was assessed using the percentage reduction in *R*^*2*^. The magnitude of the reduction in *R*^2^ is a measure of the importance of a variable [1]. As illustrated in Additional file 1: Table A2, variable x5 and x6 were the most important variables, since their removal from the model led to a reduction in *R*^2^ of about 56 and 17% respectively, while noise variables had a reduction in *R*^2^ of less than 1%. In the GBSG study, the variable x5 relates to the number of positive lymph nodes, a variable known to be the dominating prognostic factor in patients with breast cancer.

Data A250 was used to investigate in details the effects of IPs in selection of variables and functional forms in univariable and multivariable analysis. Details of the distributions and correlation structure for this subset of the data are presented in the additional file (see Table A3 and Table A4 in section A3 in Additional file 1). Using thresholds of 10 for kurtosis and 3 for skewness, we see that variable x3, x5, x6, and x7 have high kurtosis while variables x5 and x7 are highly skewed (Additional file 1: Table A3). To improve readability, understanding of concepts and results of the investigation for IPs, we used a structured approach to summarize the key issues in a two-part profile for methodological studies (see section 2 in Additional file 1).

## Results

### Univariable analysis for continuous variables

To illustrate the three steps of FSP, all *p*-values of the univariable function selection for each continuous predictor in dataset A250 were provided (Table 1). The best FP2 model was compared to the null model, a linear model, and the best FP1 model at *α* = 0.05. Variable x5 had an FP2 (0, 3) function, variable x6 had an FP1 (0) function, and variables x1 and x7 had linear terms, whereas x3 and x10 were not significant.

**Table 1:** Data A250. Univariable analysis for continuous variables. Columns 2–4 show the *p*-values for different FP tests; column 5 gives the final FP powers and indicates whether a variable was excluded; the last column shows the FP powers for the true multivariable model used to generate the data

Variable	FP2 vs. Null	FP2 vs. Linear	FP2 vs. FP1	Final selection	True model
x1	0.001	**0.165**	0.391	1	0.5, 1
x3	**0.856**	0.765	0.659	Out	1
x5	0.000	0.000	0.033	0, 3	−0.2
x6	0.002	0.046	**0.265**	0	0
x7	0.012	**0.449**	0.678	1	Out
x10	**0.135**	0.314	0.295	Out	1

*P*-values in bold indicate the first non-significant test of the FSP

There are clear discrepancies between the results of selecting a function univariably and the true functions from the multivariable model. Two variables with an effect were not selected (x3, x10), whereas one variable without an effect was selected (x7). The only “correctly” selected power term is FP1(0) for variable x6, but without related parameter estimates, power terms are not informative. One reason for the discrepant findings is the multivariable nature of the true model, which takes into account the effects of other variables in the model while deriving the outcome values. Several variables related to outcome were not included in the univariable models; thus, severe residual confounding occurred [23, 24]. This can be an important reason that univariable relationships severely mis-model the true functions. In addition, mis-modeling functions can also be attributed to the effects of IPs, specifically in relatively small sample sizes. It is important to note that if the significance level of 0.01 had been chosen for FSP, an FP1 function would have been selected for x5, the linear functions for x6, and x7 would have been excluded.

#### Diagnostic plot for single observations

Diagnostic plots of deviance differences for the three steps of FSP for each observation removed were created to illustrate the three tests of FSP and visually examine the data set for the presence of observations that alter the functional form of a selected FP model or selection of variables.

**Fig. 2:**
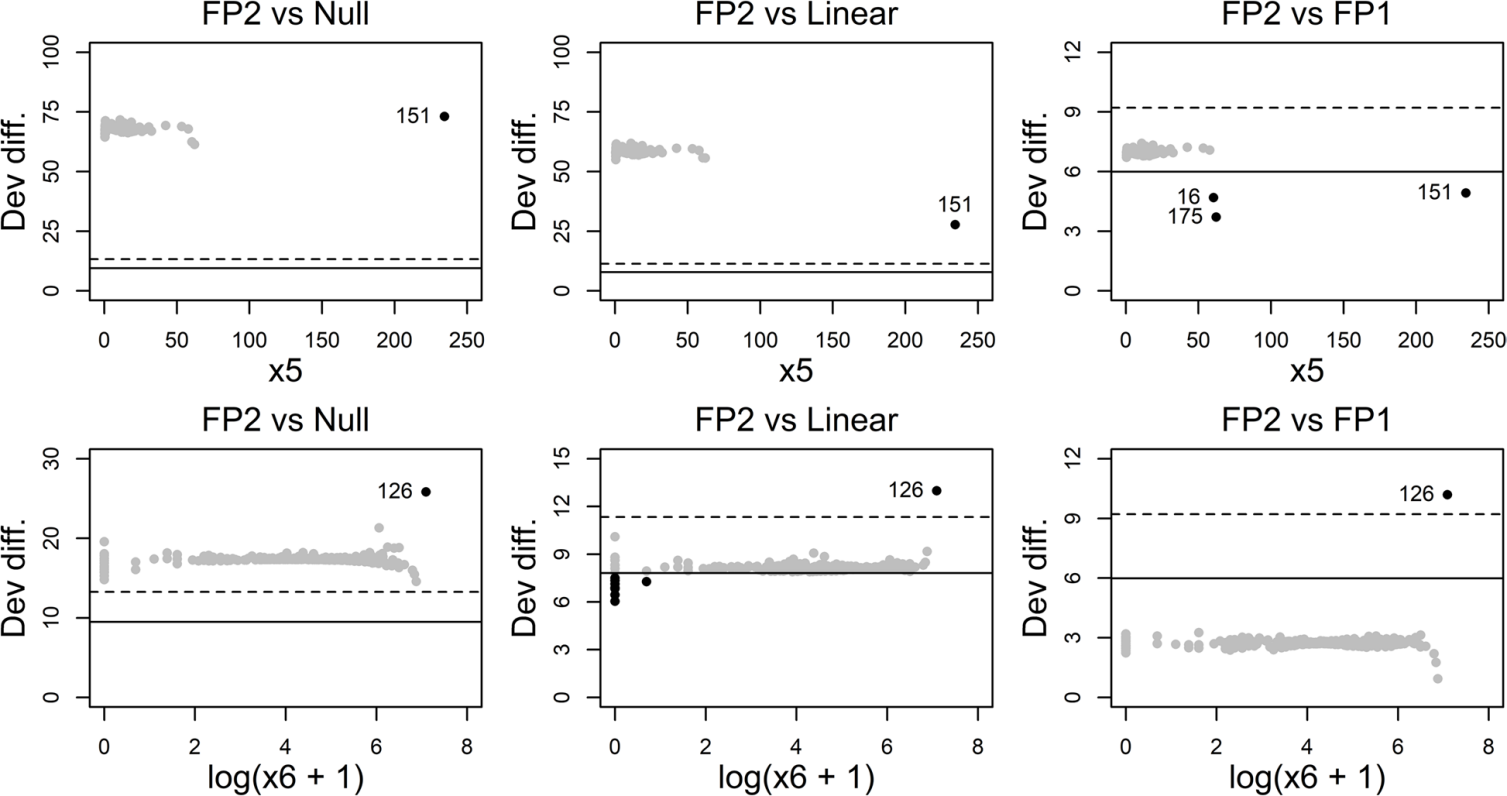
Data A250. Plots of deviance differences for each model comparison against observed values for variables x5 and x6. A logarithm scale was used for variable x6 to ease visualization due to extremely large values. Please note that *y*-axis scales differ. Two threshold values, representing the significance levels *α* = 0.05 and *α* = 0.01, are shown on the plots as horizontal solid and dashed lines, respectively. Please note that the test of FP2 vs linear and FP2 vs FP1 may not be relevant if the test of FP2 vs Null is not significant. Nevertheless, we will always show the full panel

Figure 2 shows the results of the three model comparisons for two variables (x5 and x6) with IPs. For x5, the first two FSP tests were significant, irrespective of which of the two significance levels was used. Three IPs (obs. 16, 151, and 175, shown as black dots) were identified as observations which affect the shape of the selected function for variable x5 (top right). If any of these observations were removed, the FP2 vs. FP1 test would be non-significant at the 5% level, resulting in the choice of a simpler FP1 model. Although the values of x5 for observations 16 and 175 were in proximity to other observations, the former had a larger influence on the deviance difference and is thus the first potential candidate to be eliminated from the data. In principle, we could have used a stepwise approach and eliminated one observation at a time (starting with obs. 175 because it had the largest influence or with obs. 151 because it had an outlying value for x5) before repeating the investigation with the remaining 249 observations.

To illustrate a different situation, we present results for variable x6 (lower panel of Fig. 2). The first FSP test (FP2 vs. Null) was significant at 0.05 amd 0.01 levels. Several interesting aspects were revealed in the second (FP2 vs. Linear) and third (FP2 vs. FP1) tests. First, both tests were significant at a 0.01 level when obs. 126, was eliminated. This indicated that removing this observation resulted in an FP2 function. Second, the elimination of observations other than 126 cast doubt on the need for a non-linear function because all of the deviance difference values (FP2 vs. Linear) were close to the chi-square critical value at the 0.05 level, with fewer values below the critical value, suggesting that their removal would result in the selection of a linear function.

#### Diagnostic plot for combinations of two observations

**Fig. 3:**
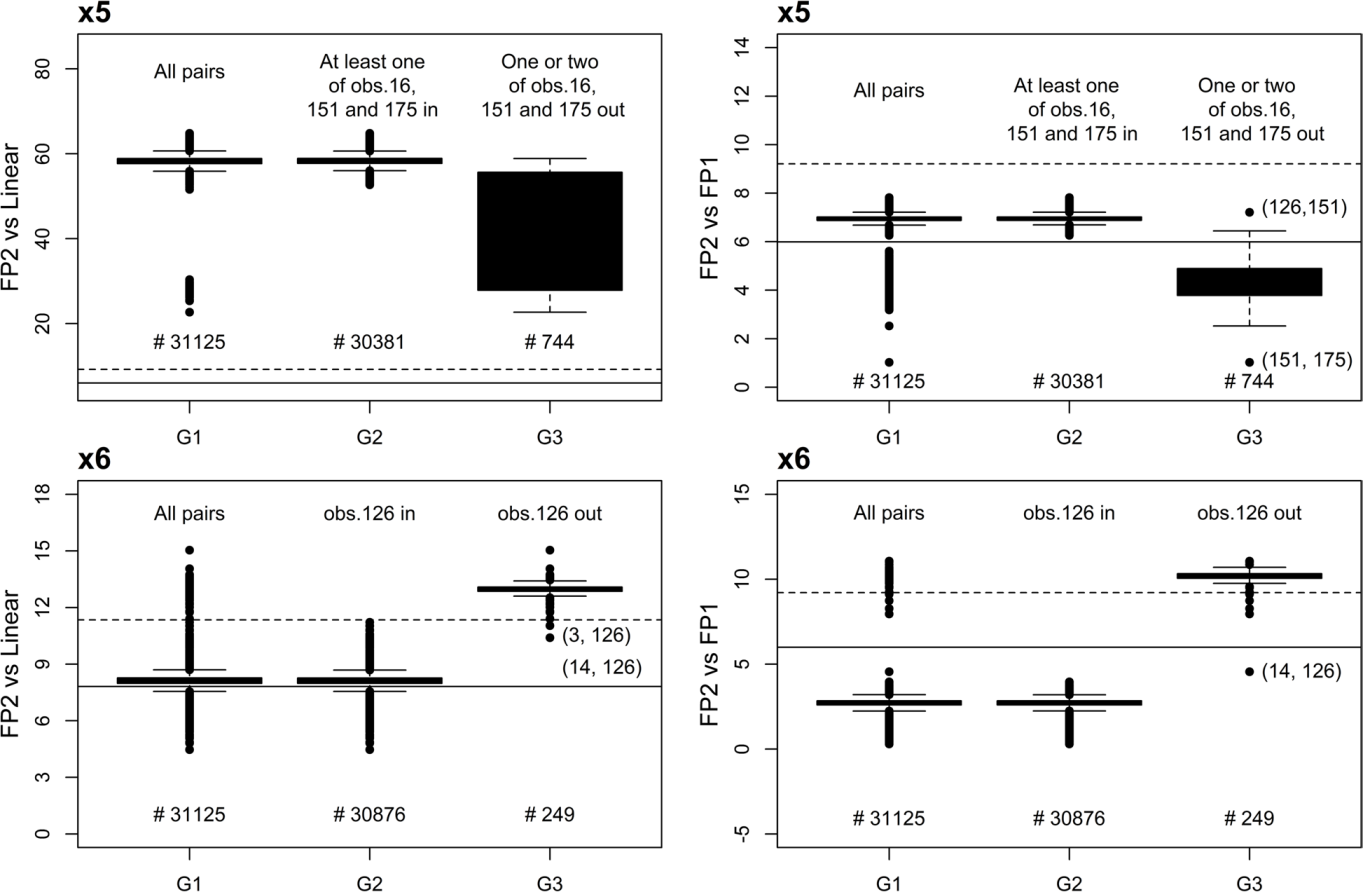
Data A250. Detection of IPs in variable x5 and x6 by deleting pairs of observations. The dashed and solid horizontal lines denote the thresholds of the FSP test at 0.01 and 0.05 level respectively. IPs are highlighted on the graph. Group G1 is the distribution of deviance difference for all 31,125 possible pairs. G2 and G3 are the distribution of pairs of subgroups, and criteria to define subgroups depend on influential points. Specific criteria are given in the figures

**Fig. 4:**
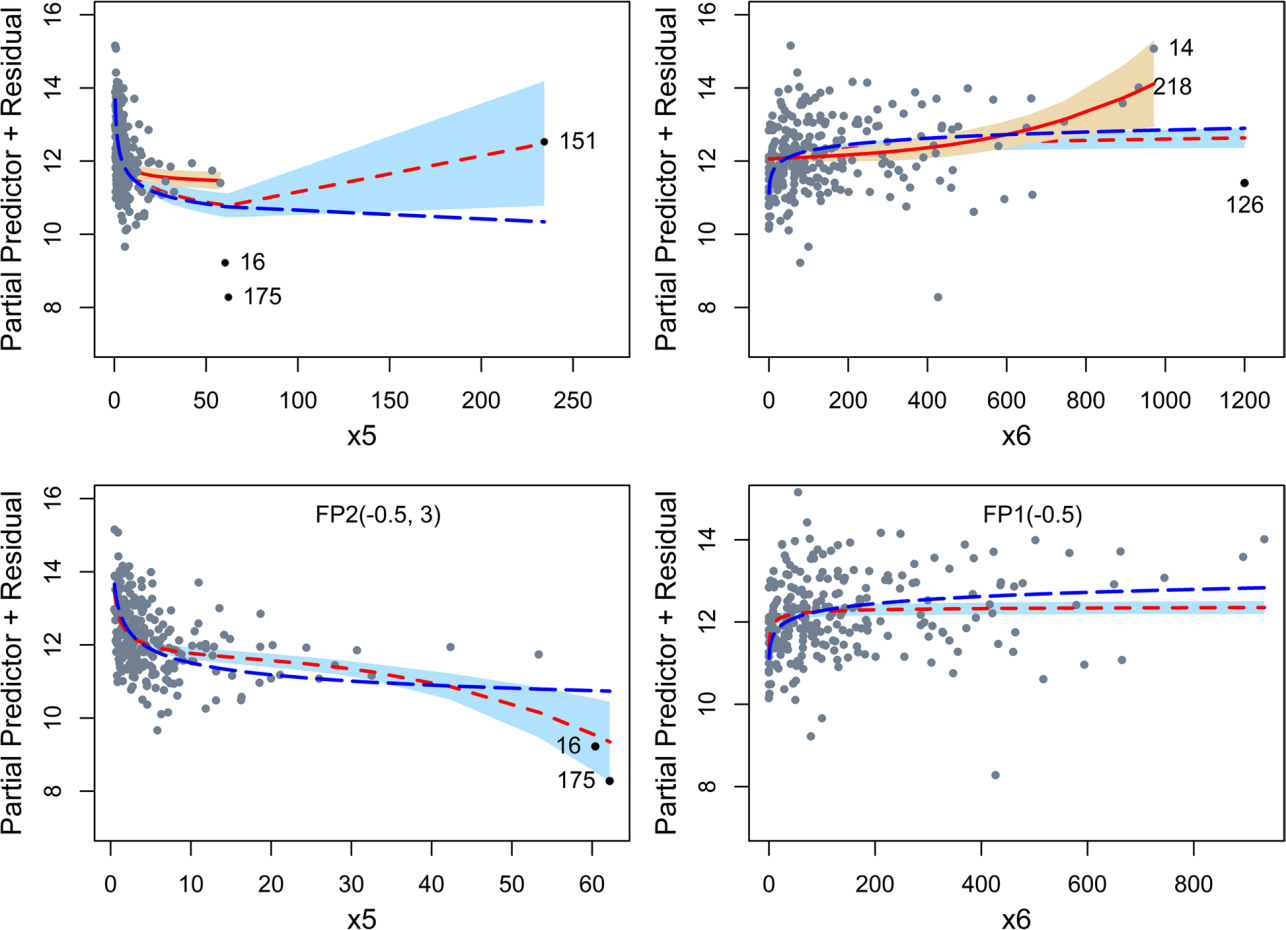
Data A250. Functional forms of variables x5 and x6. Top: the estimate of the functional form from complete data (red, short-dashed), data without IPs identified using the L-1 approach (solid line) and true function (blue, long-dashed line). Bottom: the estimate of the functional forms of variables x5 (left) and x6 (right) after the removal of observation (126, 151) and (14, 126), respectively. Please note the different scales

Deletion of pairs of observations to identify possible IPs was also conducted. Figure 3 displays the deviance differences for the last two FSP tests summarized using three groups of boxplots for variables x5 and x6 that had IPs. Group G1 shows the distribution of deviance difference for all 31,125 possible pairs. G2 and G3 are the distribution of pairs of subgroups; criteria to define subgroups depend on influential points. Specific criteria are given in the figures (Fig. 3). For variable x5 (top-left panel), two groups of deviance differences were evident, as shown in G1 under the test of FP2 vs. linear where obs. 151 was the grouping factor. The deviance difference was reduced when one or two of the obs. 16, 151, or 175 were removed (G3), but the test of FP2 vs. linear was still significant, indicating that a non-linear function was needed for x5. Similarly, in the test of FP2 vs. FP1, the groups are separated by the chi-square threshold at 5.991, indicating that the elimination of at least one of the observations 16, 151, or 175 (group G3) led to the non-significance of the test in most cases, resulting in the selection of an FP1 function. The inclusion of at least one of these three observations (group G2) led to an FP2 function for the significance level of 0.05. Deletion of pair (126, 151) resulted in the selection of an FP2 (−0.5, 3) function instead of a simpler FP1. Further scrutiny on the functional plot (bottom-left panel of Fig. 4) after the deletion of pairs (126, 151) revealed that obs. 16 and 175 were the main causes of an FP2 function. This confirms that the three observations (16, 151, and 175) were indeed influential. Deletion of these three observations produced a simpler FP1 (−0.5) function, pointing out that the complex FP2 function was not required.

For variable x6, the test of FP2 vs. FP1 (Fig. 3, bottom right) identified two groups. The second group (G2) contained all pairs with influential obs. 126. Its presence in the data resulted in the selection of an FP1 function, while its deletion resulted in an FP2 function at 0.05 level (G3). The deletion of a pair (14, 126) revealed that another observation number 14, which was not influential in single-case deletion, was influential. This explains why an FP2 function was selected when obs. 126 was deleted (Fig. 4, top right). After removal of the two IPs (14 and 126), an FP1 (−0.5) function was selected (Fig. 4, bottom-right panel). Hence, it was sufficient to describe x6 using a simpler FP1 function rather than an FP2 function.

#### Plot of functions

Figure 4 displays the functional forms of variables x5 (top-left panel) and x6 (top-right panel) before and after IP removal. There were no IPs found for the other continuous variables (x1, x3, x7, and x10). For x5, the true function FP1 (−0.2) was quite similar to the FP2 (0, 3) function from all the data up to about x5 = 50. Thereafter, there was a huge deviation due to the influence of obs. 151. The FP1 (−0.5) function obtained by omitting observations 16, 151, and 175 was a better approximation of the true function than the FP2 function estimated from all the data. The larger uncertainty (wider 95% point-wise confidence interval) towards the right end is a result of fewer observations with values of x5 larger than 50. It is important to note that the uncertainty of the function is underestimated because the function was derived data-dependently, an aspect ignored here. Furthermore, the estimated function refers to a univariable model, whereas the data were generated using a multivariable model with some correlated covariates.

The true and selected function for variable x6 with all the data was slightly different even though both functions were FP1(0) (top-right panel). The difference was caused by true and estimated coefficients (*β*_*true*_ = 0.25 and${\hat{\beta}}_{estimated}=0.15$) as well as the effects of influential obs. 126. Deletion of obs. 126 resulted in an FP2 (−1, 3) function, but closer inspection revealed that the data might contain other IPs (e.g., obs. 14 or 218).

#### Investigation of function replicability

**Fig. 5:**
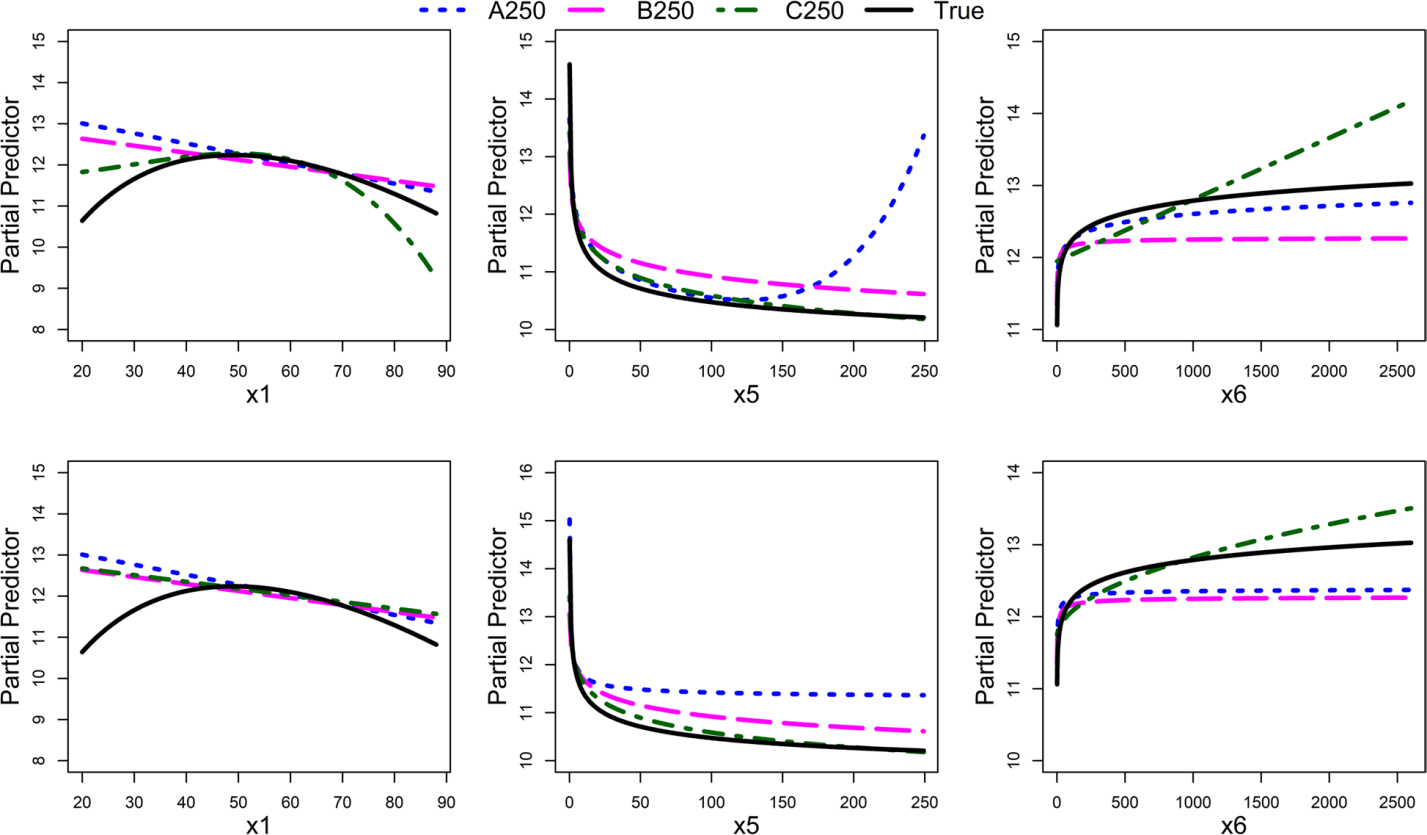
Data A250, B250, and C250. Functional forms of continuous variables in univariable analysis for x1, x5, and x6 that were selected in three datasets. Variable x10 was only selected in C250 and had a linear function, hence its plot is not provided. The upper panel shows the plots from complete data, while the lower panel shows the plots after the removal of IPs

The replicability of the selected univariable functions was investigated across three data sets (A250, B250, and C250). The functional forms of continuous variables were compared before and after IPs were removed as shown in Fig. 5 which is based on the results of Table 2. The graph of variable x5 (top-middle panel) demonstrates how an IP can lead to an unnecessary complex function. When the IP was removed (bottom-middle panel), the functional form of variable x5 was quite similar to the true function. A linear function of variable x1 did not correspond to the true FP2 function (bottom left). For variable x6, the functional forms were similar to the true function after IPs were removed as expected since this variable had a strong effect and the correlation in the data was low. These findings indicate that function replicability is influenced by both sample size and IPs. More information on identifying IPs in data B250 and C250 can be found in the additional file (see Figure A1, A2, A3, A6, and A7 in Additional file 1).

**Table 2:** Data A250, B250 and C250. Univariable analysis for continuous variables. “All data” and “all data-IPs” refer to FP powers obtained with complete data and after removing IPs, respectively. Variable (a, b, c) refers to the total number of IPs for each variable in each dataset, where a, b, and c stand for A250, B250, and C250 respectively. “=” denotes same power term selected

	A250		B250		C250		
Variable(a,b, c)	All data	All data-IPs	All data	All data-IPs	All data	All data-IPs	True
x1(0, 0, 1)	1	=	1	=	3, 3	1	0.5, 1
x3(0, 0, 0)	Out	=	Out	=	Out	=	1
x5(3, 0, 2)	0, 3	−0.5	0	=	0	0.5, 0.5	−0.2
x6(2, 0, 1)	0	−0.5	−0.5	=	1	0.5	0
x7(0, 3, 0)	1	=	Out	0	0	=	Out
x10(0, 0, 0)	Out	=	Out	=	1	=	1

### Multivariable analysis—effect of influential points

#### Elimination of influential points identified in univariable analysis

MFP analyses were run to generate multivariable models for data A250, B250, and C250 before the IPs were deleted. The selected models are displayed in Table 3, in the column labeled “all”. Next, all the IPs identified in univariable analyses for the six continuous variables were deleted and an MFP model was fitted, the results of which are displayed in the column labeled “IPXu.” Finally, the column labeled “IPXm” presents the MFP model selected after deleting IPs that were identified in the diagnostic analysis of the multivariable model.

**Table 3:** Data A250, B250, and C250. Selected MFP models with complete data (“all”) and after removal of IPs identified from the univariable (IPXu) and multivariable (IPXm) diagnostic analyses. The number of IPs identified univariable and multivariable, respectively, are shown in parentheses. “=” is used if the power selected agreed to the power from all data

Type	Variable	Dataset A250 (5, 5)			Dataset B250 (3, 3)			Dataset C250 (4, 6)			True model
		All	IPAu	IPAm	All	IPBu	IPBm	All	IPCu	IPCm	
Cont.	x1	1	=	=	−1, 3	0, 3	1	Out	=	=	0.5, 1
	x3	1	=	=	Out	=	=	Out	=	=	1
	x5	0, 3	−0.5	0	0	=	=	−0.5	=	=	−0.2
	x6	0	=	=	−0.5	=	=	0	=	=	0
	x7	Out	=	=	Out	=	=	Out	=	=	Out
	x10	1	=	3	Out	=	=	1	=	Out	1
Cat.	x9a	Out	=	=	In	=	=	Out	=	=	Out
	x9b	Out	=	=	Out	=	=	Out	=	=	Out
Bi	x2	Out	=	=	Out	=	=	In	=	=	Out
	x4a	In	=	=	In	=	=	In	=	=	In
	x4b	Out	=	=	Out	=	=	Out	=	=	Out
	x8	In	=	=	Out	=	In	In	=	=	In

Cont, Cat, and Bi denote continuous, categorical, and binary variables respectively

In the univariable analysis, a total of 5, 3, and 4 IPs were identified in A250, B250, and C250, respectively. Deleting these observations resulted in the selection of variables similar to the model fitted to the full data. However, in data A250, a simpler FP1 (−0.5) function was estimated for variable x5 after deleting IPs rather than an FP2 (0, 3) function from complete data. In data set B250, different powers of FP2 functions were also estimated for variable x1. Compared to the results from the univariate investigations (Table 2), several functions differ substantially. For x1, a linear function was selected in B250 whereas an FP2 is selected with the multivariable approach (all and IPBu). In A250, x3 was not significant in the univariate analyses but was included with a linear function in the multivariable case.

#### Diagnostic analyses on multivariable model

Diagnostic analyses were performed on the selected multivariable model (column “all” in Table 3) as a second way to check for IPs in a multivariable context. The IP investigation for dataset A250 is described in this section, while the IP investigations for datasets B250 and C250 were described in the additional file (see section A4 in Additional file 1).

In leave-one-out approach (Figure A4 Additional file 1), obs. 175 was found to influence the functional form of variable x5 at the 0.05 level. Its removal turned an FP2 (0, 3) function into an FP1 (−0.5) function. In the leave-two-out approach, IPs were found in variables x5 and x10. For variable x5, deletion of any pair with obs. 175 rendered the test of FP2 vs. FP1 non-significant except when two pairs (37, 175) and (151, 175) were deleted (Additional file 1: Figure A5). An inspection of the functional forms (Figure A5 Additional file 1) revealed that when a pair (37, 175) was deleted, an FP2 function was estimated because of the effects of obs. 151 that was still in the data. Similarly, when a pair (151, 175) was deleted, an FP2 function was driven by obs. 37. As such, observations 37, 151, and 175 were indeed influential in variable x5. An easy and informal way to check for the three IPs simultaneously is by deleting three observations at a time instead of pairs. Only two observations, 151 and 175, were influential in both univariable and multivariable checks for IP. For variable x10, deleting two pairs (37, 76) and (74, 76) rendered the test of FP2 vs. linear significant, implying that observations 37, 74, and 76 were IPs. Deleting any of the pairs led to an FP1 function. In total, five IPs (37, 74, 76, 151, and 175) were identified in A250 as presented in Table 3.

**Fig. 6:**
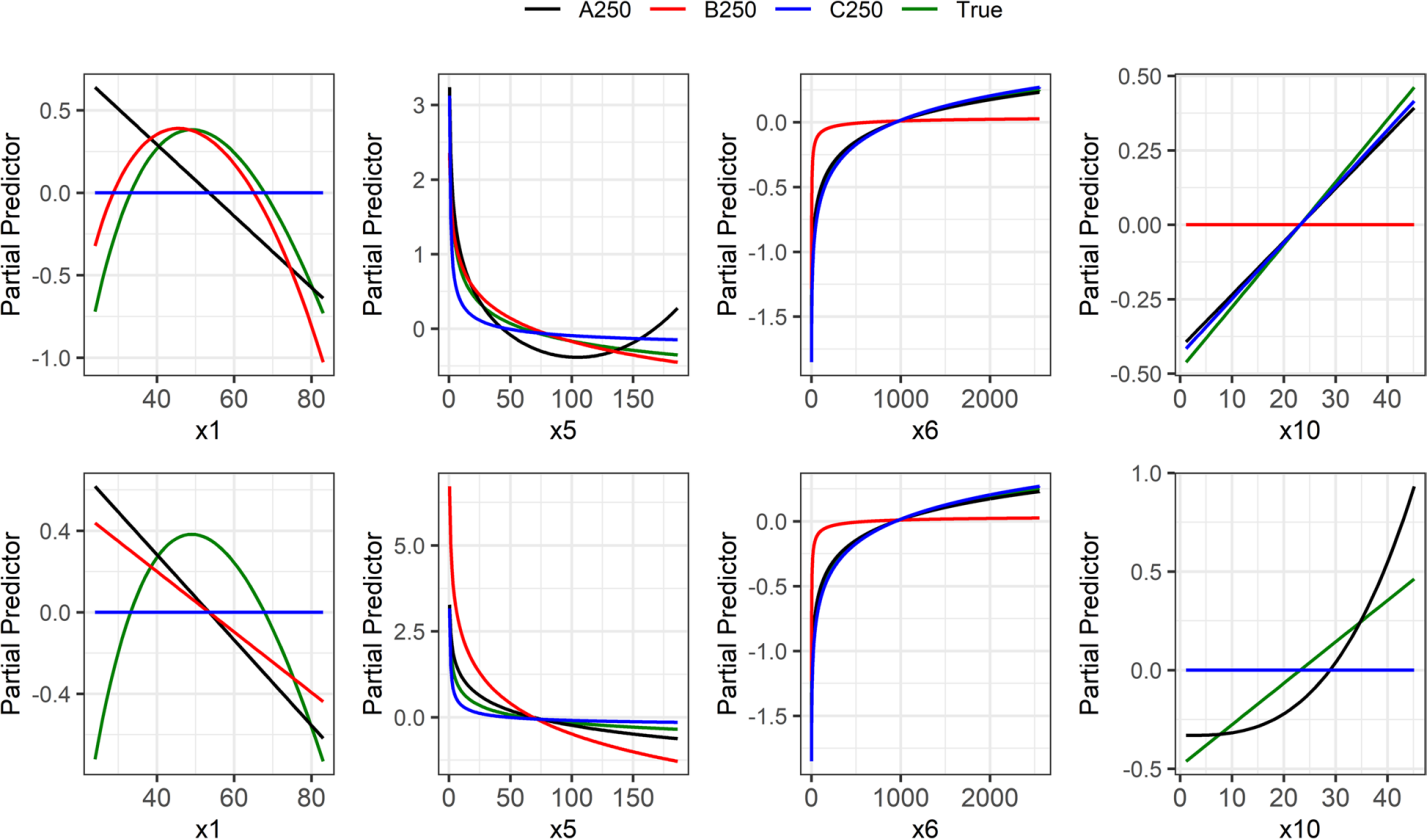
Data A250, B250, and C250. Functional forms of continuous variables for the selected MFP models (see Table 3). The upper panel shows the plots from complete data, while the lower panel shows the plots after the removal of IPs. The horizontal line indicates that no variable was selected. Not shown are x3 (linear in true and A250, out in B250 and C250) and x7 (true out and never selected).

Table 3 compares models from complete data (“all”) and after eliminating IPs (IPAu and IPAm) in the three datasets with a sample size of 250. Elimination of IPs had an influence on some of the selected functions (x1 in B250, x5 in A250, and x10 in A250 and C250). IPs had also an influence on the selection of the binary variable x8 in B250. In particular, in A250 an FP2 (0, 3) function was estimated for variable x5 due to the effects of IPs and a satisfactory function is FP1 (0) which was quite similar to the true function (Fig. 6). However, elimination of IPs may also result in the selection of a non-linear function instead of a linear function (x10 in A250).

More important is the comparison of the selected models with the true model. Concerning the inclusion of binary and categorical variables, we observed full agreement in A250, a difference for x2 in C250, and some differences in B250. Concerning power terms of functions, we observed good agreement for x6 and x7 (was always out), and non-linear functions selected for x5 in all analyses. Several disagreements were observed in other variables. In specific, for x1, where FP2 was the true function, but the variable was excluded in C250 and a linear function was estimated in A250, a strong indication that the power was insufficient to identify the non-linear effect. A larger sample size seems to be needed.

Figure 6 compares the functional forms of continuous variables from three datasets with and without IPs. The true function for variable x1 was an FP2, which was well approximated by data B250 before the removal of IPs but elimination of IPs resulted in the selection of a linear function which is far away from the true effect of x1.The true and estimated functions for variables x5 and x6 were nearly identical when IPs were removed.

### Sample size and its effect on identifiability of the true model

To evaluate the effect of the sample size on the identifiability of the models, we compared models derived with different sample sizes and also after IPs were deleted. Univariable and multivariable approaches were used to check for IPs.

#### Small to relatively large dataset

**Fig. 7:**
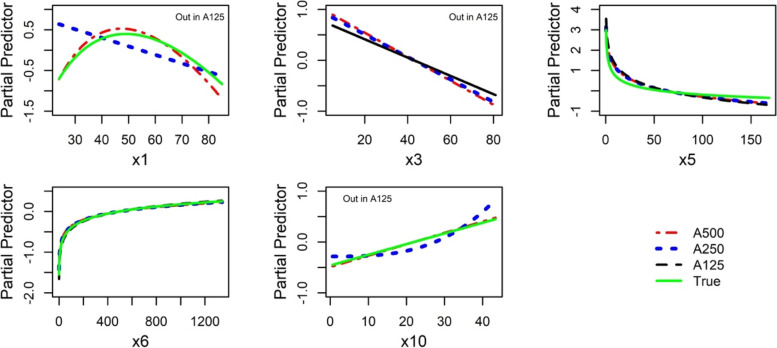
Data A125, A250, and A500. Functional forms of continuous variables after elimination of IPs identified in multivariable model (results of IPAm in Table 4). Variables x1, x3, and x10 were not selected in A125

Table 4 summarizes the power terms of the nine models selected from small to relatively large datasets, while Fig. 7 shows related functions for data without IPs (i.e., IPAm).

**Table 4:** Data A125, A250, and A500. Selected functions from MFP models with all data (“all”) and after removal of IPs identified from the univariable (“IPAu”) and multivariable (“IPAm”) diagnostic analyses. The number of IPs identified in each respective analysis is shown in parentheses next to the name of the data

Type	Variable	Data A125 (3, 0)			Data A250 (5, 5)			Data A500 (6, 1)			True model
		All	IPAu	IPAm	All	IPAu	IPAm	All	IPAu	IPAm	
Cont.	x1	Out	=	=	1	=	=	0.5, 1	=	=	0.5, 1
	x3	Out	1	Out	1	=	=	1	=	=	1
	x5	0	−0.5	0	0, 3	−0.5	0	0, 3	−1, 0	0	−0.2
	x6	0	=	=	0	=	=	0	=	=	0
	x7	Out	=	=	Out	=	=	Out	=	=	Out
	x10	Out	=	=	1	=	3	1	=	=	1
Cat.	x9a	Out	=	=	Out	=	=	Out	=	=	Out
	X9b	Out			Out	=	=	In	=	Out	Out
Bi.	x2	Out	=	=	Out	=	=	Out	=	=	Out
	x4a	Out	=	=	In	=	=	In	=	=	In
	x4b	Out	=	=	Out	=	=	Out	=	In	Out
	x8	In	=	=	In	=	=	In	=	=	In

Multivariable analysis of the complete data set A125 led to the selection of only three variables: x5, x6, and x8 due to low power for selecting variables with moderate effects (Table 4). Even though the sample size was relatively small, non-linearity of x5 and x6, the two variables with a stronger effect (see Additional file 1: Table A2), was identified. The removal of three IPs (obs.14, 16, and 105) that were identified in the univariable approach led to the inclusion of variable x3 and changed the FP1 power term for variable x5. No IPs were found in the diagnostic analysis of the multivariable model of data set A125. Compared to the true model, the main differences in selected MFP models were the elimination of x1, x10, and x4a, while x3 was only included with the IPu approach. These results illustrate that the sample size of 125 was much too low to select a suitable MFP model.

The results for the sample size of *n* = 250 were much closer to the true model since variables x1 (although only linear), x3, x10, and x4a were included in the model. The elimination of five IPs did not affect the selection of variables but changed some of the power terms of continuous variables. For *n* = 500, the selected MFP models agreed well to the true model. Selected functions for x1 (Fig. 7) best illustrate the significant impact of the sample size. The variable was eliminated when *n* = 125, a linear function was selected when *n* = 250, and an FP2 function that was close to the true function was selected when *n* = 500.

#### Relatively large dataset

**Fig. 8:**
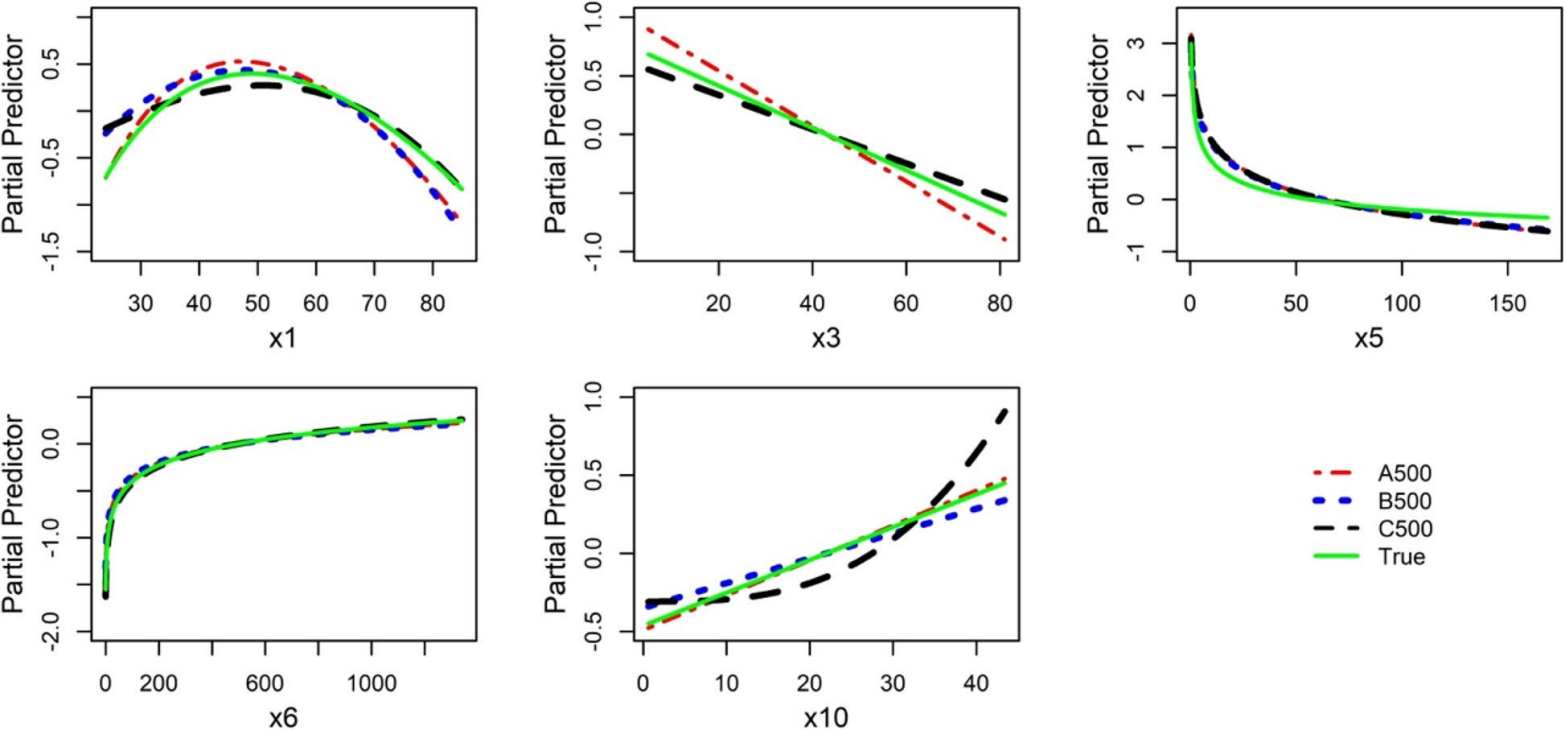
Data A500, B500, and C500. The plots were created after removing the IPs identified in the multivariable model (results of IPAm in Additional file 1: Table A5). Variable x7, which was irrelevant in the true model, was not selected in each data set, so it was not plotted

Results for three relatively large datasets (A500, B500, and C500) were summarized in the additional file (see subsection 4.3 in Additional file 1). IPs had some effects on the power terms chosen, and binary variables were not always correctly included. Figure 8 shows the estimated functions (after deletion of IPs) for the five continuous variables that had an effect on the outcome. In C500, a non-linear function was estimated for variable x10 instead of the correct linear function but otherwise the agreement is good. Identification and elimination of IPs improved the selected function for x5 (FP2 in all data, FP1 after removal of IPs) and changed the selection of x9b and x4b in A500, but otherwise the effect was negligible in the three data sets.

One of the reviewers suggested to investigate the effects of IPs in larger dataset which is often encountered in practice. This prompted us to conduct additional analysis in data D1000 with a sample size of 1000. Due to computational complexity, we only conducted a single-case deletion and the multivariable approach. No IPs were found at the 5% significance level for function selection, but three IPs were found at 1% in only variable x5 which caused an FP2 function as shown in Additional file 1: Figure A8. The FP2 function was clearly driven by IPs (Additional file 1: Figure A9, left panel). The function without IPs agreed well with the true function (Additional file 1: Figure A9, right panel).

Generally, with large sample sizes and removal of IPs, variables selected and the estimated functional forms were good approximations of the true model (Additional file 1: Table A5).

## Discussion

In areas of science in which empirical data are analyzed, various types of regression models are derived for prediction, description, and explanation [25]. In medicine, continuous measurements such as age and weight are often used to assess risk, predict an outcome, or select a therapy. Background knowledge or the type of question should strongly influence how continuous variables are used. However, knowledge is often insufficient and the analyst needs to decide how to handle continuous variables, a very difficult issue in the context of multivariable analysis when the selection of the functional form of a continuous variable needs to be combined with the selection of variables which have an influence on the outcome.

Concerning continuous variables, categorization and the assumption of a linear effect are still the most popular approaches [26], despite many well-known weaknesses [2–4, 13]. This unfortunate situation is partly caused by lack of guidance for the selection of variables and modeling of continuous variables. Sauerbrei et al. [13] described and discussed the fractional polynomial and spline-based approaches in an overview paper of topic group 2 “Selection of variables and functional forms in multivariable analysis” of the STRengthening Analytical Thinking for Observational Studies (STRATOS) initiative [27]. Various spline-based approaches have been proposed and an overview of the most widely used spline-based techniques and their implementation in R software is given in [12]. The authors illustrated some challenges that an analyst face when working with continuous variables using a series of simple scenarios of univariable data. They concluded that an “…experienced user will know how to obtain a reasonable outcome, regardless of the type of spline used. However, many analysts do not have sufficient knowledge to use these powerful tools adequately and will need more guidance.”

Univariable analysis was the emphasis of this overview. A brief overview of spline-based techniques for multivariable model building was given in [13]. While FPs are global functions, splines are much more flexible and can also estimate local effects. However, that comes at the price of more function instability and uncertainty [7]. Furthermore, local features may be identified by a systematic check of residuals of the MFP model, and statistically significant local polynomials can be parsimoniously added [28]. Results of MFP and spline-based approaches were compared in several examples [1, 7], and a simulation study [29], but it is obvious that more comparisons of spline procedures in both univariable and multivariable contexts and comparisons to MFP are needed.

In contrast to the spline approaches, the MFP procedure is a well-defined pragmatic approach. Deriving suitable models for description is the main aim, and the two significance levels for the BE and FSP parts are the key tuning parameters. Using simulated data, we illustrated all steps of the procedure and the importance of checking whether IPs affect (strongly) the selected model with the potential consequence of (severe) errors in variables or functional forms selected. IPs can also have a strong effect on model (in-)stability [17]. Leave-one-out and leave-M-out are simple and helpful techniques for the identification of IPs which can be easily understood by most analysts with at least some background in regression modeling. It is important to check each multivariable model that includes continuous variables for potential IPs. Here, we eliminated identified IPs, but other options may be preferable in real data.

The effects of sample size on MFP models were illustrated in datasets A125, A250, and A500 (Table 4 and Fig. 7). We observed that MFP models derived from a relatively small sample size (A125) deviated severely from the underlying true model since some relevant variables were excluded and linear functions were estimated for some continuous variables instead of non-linear functions, probably due to low power to detect non-linearity [1]. We also observed that an MFP can detect stronger non-linear functions in small sample sizes (e.g., variables x5 and x6). As the sample size increased (A500), the performance of MFP improved drastically since important variables were correctly selected and non-linear functions (e.g., x1 and x3) were identified. In addition, all models derived with a relatively large sample size (500 observations, 12 variables, about 42 observations per variable) and IPs eliminated were similar to the true model as shown in Additional file 1: Table A5 and Fig. 8. These results indicate that with about 50 or more observations per variable, it may be possible to derive suitable descriptive models for studies with several variables ranging from about 5 to 30. In our simulated data, we had six continuous and six binary variables.

The results of the function selection procedure can be driven by IPs. For instance, the estimated functional form for variable x5 (Fig. 4) from the complete data with IP is a non-monotonic FP2 function instead of a monotonic FP1 function. Similar results were observed in the case study where an FP2 function was estimated for the variable abdomen instead of a linear function (Additional file 1: Table A6). These results indicate that the data analyst needs to use the algorithm carefully while selecting the functional forms of continuous variables since, in some instances, a simple function may suffice instead of a complex function driven by IPs (see Additional file 1: Table A7 and Figure A10). Plots of deviance differences for variables x5 and x6 (Figs. 2 and 3) illustrate that such additional investigation can support the final decision for a model, e.g., we might prefer a simpler model despite a (just) significant result for the more complex model. Comparisons of two competing functions (e.g., linear versus best FP1) may show that the difference is small and subject matter knowledge or practical usefulness may be used as a criteria for the final selection.

As often done, we started with the investigation of one variable, while our outcome was created according to a multivariable process. Such marginal investigation may be misleading, and researchers may prefer to derive a multivariable model and check whether single points have a severe influence on the model selected. In several datasets, we conducted such an approach and found some differences in potential IPs identified. We did not check whether variables eliminated by MFP would have been included if we had eliminated single observations from the data set. In real data, we would recommend that. If a single continuous variable is of main interest (e.g., a continuous risk factor in epidemiology), it is straightforward to use our “univariable” investigations, adjusted for relevant confounders, to check whether single points drive the selected function for this variable.

## Conclusions

Variable selection by using backward elimination and the fractional polynomial function selection procedure can be easily understood and used by non-experts. It is obvious that the sample size needs to be sufficient and aspects of model criticism should be standard for each derived multivariable model. We concentrated on the importance of IPs, but other aspects (e.g., residual plots) are also relevant. Some issues are discussed in chapters 5, 6, and 10 in [1], and on the MFP website. If the effect of continuous variables needs to be investigated in the context of a multivariable regression model, recommendations for practice were proposed under several assumptions ([1] Chapter 12.2, 7).

If the sample size is too small, models selected with the MFP approach might differ substantially from the underlying true model. However, for larger sample sizes, a carefully conducted MFP analysis is often a suitable way to select a multivariable regression model which includes continuous variables. In such a case, MFP can be the preferred approach to derive a multivariable descriptive model.

## Supplementary Information

**Additional file 1: Table A1.** MethProf-simu profile giving an overview of the aims, data, estimand or target of analysis, methods and performance measures (ADEMP structure) in part A. All analyses are listed in part B, categorized into analysis (A), presentation (P) and description of data (D). **Table A2.** ART data (N = 5,000, *R*^*2*^=0.49). Contribution of each predictor to the model fit, expressed in terms of the percentage reduction in *R*^*2*^ when regressing the index on all predictors minus the one of interest. The last column shows the variables that was used to generate the outcome variable. **Table A3.** Data A250. Descriptive statistics for continuous (top) and categorical (bottom) variables. **Table A4.** Data A250. The entries above and below the main diagonal are Spearman correlation coefficients with absolute values larger than 0.25 and differences between Spearman and Pearson correlation coefficients greater than 0.05 for continuous variables. **Figure A1.** Data C250. Identification of influential points in univariable analysis using leave-one-out approach. **Figure A2.** Data C250, univariable analysis. Smoothed residuals with 95% pointwise confidence intervals for variable x5 and x6 before and after removal of IPs. **Figure A3.** Data C250. Functional form of variable x7 in full data (dashed line) and without observation 104 (solid line). Truncated at 600. **Figure A4.** Data A250. Identification of influential points using L-1 approach in the selected MFP model (see Table 3, all data). **Figure A5.** Data A250. Identification of influential points in multivariable analysis using leave-two-out approach. left panel: functional form for x5 when the pair (37, 175) was removed. Right panel: functional form for x5 when pair (151, 175) was removed. **Figure A6.** data B250. Identification of influential points in the selected MFP model (see Table 3). Multivariable analysis using L-1 approach. **Figure A7.** data C250. Identification of influential points of x10 in the selected MFP model using L-1approach. **Table A5.** Data A500, B500, C500 and D1000. Multivariable analysis for relatively large datasets. See Fig. 8, A8 and A9 for the related functions. **Figure A8.** data D1000. Identification of influential points in the selected MFP model (see Table A5). Multivariable analysis using L-1 approach. No IPs identified at 5% level, but 3 IPS identified at 1% in variable x5. **Figure A9.** Data D1000. Identification of influential points in multivariable analysis using L-1. left panel: functional form for x5 in full data. Right panel: functional form for x5 before (red solid line) and after (blue dashed line) removal of observations 379, 664 and 925. The green solid line is the true function. **Table A6.** Data body fat, univariable analysis. *P*-values for different model comparison are displayed in column 2-4. The last two columns show the FP powers or exclusion of a variable in the complete data set and after deleting influential points respectively. **Table A7.** Data body fat. Selected models from a MFP analysis with all data represented by MFP(0.05, 0.05) and after removal of IPs identified from univariable (IPBFu (k)) and multivariable (IPBFm(l)) diagnostic analyses where k and l represent the number of IPs identified in the corresponding analysis. MFP (1, 0.05) – no elimination of variables, FSP with significance level 0.05. **Figure A10.** Data body fat. Multivariable analysis of complete data. Functional forms for continuous predictors in MFP (0.05, 0.05) model. Deleting 3 IPs biceps is no longer significant.

## Data Availability

To encourage understanding of MFP methodology, we will make all programs available with the published manuscript. All steps of our investigations can be replicated using the data accessible on the book’s website (https://www.uniklinik-freiburg.de/imbi/stud-le/multivariable-model-building.html). The corresponding R code with examples will be provided at https://github.com/EdwinKipruto/mfp-influential-points.
