# Supplementary material for: Effects of influential points and sample size on the selection and replicability of multivariable fractional polynomial models
Source: Diagn Progn Res. 2023 Apr 18;7:7. doi: 10.1186/s41512-023-00145-1 (PMC10111698; doi:10.1186/s41512-023-00145-1)
Supplement: Supplementary file 1 — Additional file 1: Table A1. MethProf-simu profile giving an overview of the aims, data, estimand or target of analysis, methods and performance measures (ADEMP structure) in part A. All analyses are listed in part B, categorized into analysis (A), presentation (P) and description of data (D). Table A2. ART data (N = 5,000, R2=0.49). Contribution of each predictor to the model fit, expressed in terms of the percentage reduction in R2 when regressing the index on all predictors minus the one of interest. The last column shows the variables that was used to generate the outcome variable. Table A3. Data A250. Descriptive statistics for continuous (top) and categorical (bottom) variables. Table A4. Data A250. The entries above and below the main diagonal are Spearman correlation coefficients with absolute values larger than 0.25 and differences between Spearman and Pearson correlation coefficients greater than 0.05 for continuous variables. Figure A1. Data C250. Identification of influential points in univariable analysis using leave-one-out approach. Figure A2. Data C250, univariable analysis. Smoothed residuals with 95% pointwise confidence intervals for variable x5 and x6 before and after removal of IPs. Figure A3. Data C250. Functional form of variable x7 in full data (dashed line) and without observation 104 (solid line). Truncated at 600. Figure A4. Data A250. Identification of influential points using L-1 approach in the selected MFP model (see Table 3, all data). Figure A5. Data A250. Identification of influential points in multivariable analysis using leave-two-out approach. left panel: functional form for x5 when the pair (37, 175) was removed. Right panel: functional form for x5 when pair (151, 175) was removed. Figure A6. data B250. Identification of influential points in the selected MFP model (see Table 3). Multivariable analysis using L-1 approach. Figure A7. data C250. Identification of influential points of x10 in the selected MFP model using L-1approac [file 41512_2023_145_MOESM1_ESM.docx]

**Appendix**

**Effects of influential points and sample size on the selection and replicability of multivariable fractional polynomial models**

Willi Sauerbrei^1*^, Edwin Kipruto^1*^, James Balmford^1+^,

^1^Institute of Medical Biometry and Statistics, Faculty of Medicine and Medical Center -University of Freiburg, Germany

*Joint first authorship; ^+^ deceased

1 Function Selection Procedure

As a first step in the function selection procedure (FSP), 44 regression models are fitted, each with a different FP1 or FP2 function applied to the continuous covariate in question. The deviance of each fitted model is determined, and using deviance differences the best-fitting FP1 and FP2 functions are selected.

The FSP then continues in 1, 2 or 3 steps as follows:

1. Test the best FP2 model for *x* at the α significance level against the null model using a test with four degrees of freedom. If the test of deviance difference is not significant, it should be stopped, and concluded that the effect of x is not significant at the α level, implying that the variable should not be included in the model. Otherwise it should be continued.
2. Test the best FP2 for *x* against the linear function at the α level using a test with three degrees of freedom. If the test is not significant, stop, and conclude that *x* is best modelled by a linear relationship with the response. Otherwise continue.
3. Test the best FP2 for *x* against the best FP1 at the α level using a test with two degrees of freedom. If the test is not significant, the final model is the best FP1, otherwise the final model is the best FP2. End of procedure.

2 Two-Part Structured Approach

To improve readability, understanding of concepts and results of the investigation for IPs, we used a structured approach to summarize the key issues in a two-part profile for methodological studies. Part A follows the ADEMP structure that was recently proposed by Morris et al. [1] to improve understanding and interpretation of simulation studies. Part B provides a summary of all analyses as proposed in the explanation and elaboration paper of the REMARK reporting guidelines for tumor markers and extended in a case study on the development and assessment of prediction models [2, 3]. Table A1 provides an overview of the main aim of the study, data used, and analyses conducted. We analyzed seven subsets of an artificial data set and illustrated the methods in a real data set. As proposed in the REMARK profile, the second part provides an overview of all analyses conducted and distinguishes between data description (D), analyses (A), and presentations (P). Here, investigation of model assumptions was not carried out, an important issue that should be conducted in general. In total, 21 analyses were conducted, often with parts a and b. This notation was used to show that the same analyses were conducted with leave-one-out or leave-two-out, in different datasets or in univariable or multivariable analyses. The results/remarks section points to the specific table or figure.

**Table A1:** MethProf-simu profile giving an overview of the aims, data, estimand or target of analysis, methods and performance measures (ADEMP structure) in part A. All analyses are listed in part B, categorized into analysis (A), presentation (P) and description of data (D).

**Part A ADEMP structure**

| **A**ims | - To investigate whether IPs exist and have an influence on the selected FP functions in univariable and multivariable fractional polynomial (MFP) models. - To investigate replicability of MFP models - To investigate the effects of sample size on selecting a ‘suitable’ MFP model |
| --- | --- |
| **D**ata | - We used the ART data set published on the website <http://mfp.imbi.uni-freiburg.de/book>. The data simulated 5000 patients in a normal-error regression setting with 10 predictors; six of which are continuous (x1, x3, x5-x7, x10), two are binary (x2, x8) and one each (x4 and x9) are ordinal and nominal respectively. The design was influenced by a real Germany breast cancer study. - We considered seven subsets of the data with different sample sizes abbreviated as: **A125**(obs.1-125), **A250**(obs.1-250), **A500**(obs.1-500), **B250**(obs.2001-2250), **B500**(obs.2001-2500), **C250**(obs.3001-3250) and **C500**(obs.3001-3500) - We also analysed the body fat data [4] where the outcome and predictors ($p=13)$were all continuous and the sample size $n = 252$ observations |
| **E**stimand/target of analysis | The targets: (i) investigate effects of single and pairs of observations on the deviance of models relevant for the FSP strategy (best FP2 vs. Null, best FP2 vs. Linear, best FP2 vs. best FP1), (ii) assess replicability by comparing models selected in different data sets with the same sample size and derived from the same true model and (iii) to compare models derived with different sample sizes. |
| **M**ethods | - Function selection procedure (FSP) to select FP functions - Leave-d-out­-approach (d =1 and 2) to identify IPs - In multivariable analyses we used two approaches  1. Eliminate ‘univariable’ IPs followed by MFP on reduced data 2. MFP on all data followed by check for IPs in selected model  - The MFP (α_1_, α_2_) procedure was used with $\alpha_{1}=\alpha_{2}= 0.05$ for function and variable selection. Critical value for $\alpha_{2}=0.01$was also shown to discuss some issues. |
| **P**erformance measures | - Comparisons of the true functions and the true models with functions derived with FSP and models derived with MFP for all data and after elimination of IPs.  - Use of graphical comparisons for functions. Comparisons are conducted for different data with the same sample sizes and for varying sample sizes. |

**Part B Overview of analyses and presentations**

| **Analysis** | **Dataset** | **Variables considered** | **Remarks/results** |
| --- | --- | --- | --- |
| **P1:** FP functions | NA | NA | **Fig 1.** Schematic diagram of eight FP1 and five FP2 functions. |
| **D1**: description of data | ART | y, x1-x10 | **Table A2** Variable importance based on *R^2^* |
| **D2**: description of data | A250 | y, x1-x10  as x6 has values ‘0’ we use x6+1 | **Table A3**. Distribution of variables; x5, x6 and x7 have high kurtosis **Table A4**. Correlation structure. Larger difference between Pearson and Spearman presented |
| **A1**: Function Selection Procedure (FSP); Univariable analysis | A250 | y, x1, x3, x5-x7, x10 | **Table 1**. FSP (0.05) x5 and x6 were non-linear |
| **A2a**: check for IPs in univariable analysis; (L-1) | A250 | y, x1, x3, x5-x7, x10 | **Fig 2.** x5 and x6 had IPs. Data not shown **(DNS)** for Investigation of IPs in x1, x3, x7, and x10. No IPs found. |
| **A2b**: check for IPs in univariable analysis; (L-2) | A250 | y, x1, x3, x5-x7, x10 | **Fig 3**: x5 and x6 had IPs. **DNS**: no IPs identified in other variables. |
| **P2**: effects of IPs identified in A2 on functional forms | A250 | x5, x6 | **Fig 4**. Functional forms for x5 and x6 with and without IPs identified in **A2a** and **A2b** |
| **A3a**, **A3b** as in A2a, A2b | B250 | y, x1, x3, x5-x7, x10 | **DNS.** IPs were only found in x7 |
| **A4a**, **A4b** as in A2a, A2b | C250 | y, x1, x3, x5-x7, x10 | **DNS**. IPs were found in x1, x5, x6 and x7 |
| **P3**: effects of IPs identified in A4 on model fit | C250 | x5, x6 | **Fig A1** and **A2**. Two and one IPs identified in x5 and x6 respectively, confirmed by residual plots. |
| **P4**: effects of IPs identified in A4a and A4b on functional forms | C250 | x7 | **Fig A3**. IPs introduced a hook in the function |
| **A5:** functions selected in univariable analysis; full data and data without IPs identified in **A2**, **A3** and **A4** | 250 (A/B/C) | y, x1, x3, x5-x7, x10 | **Table 2.** x5 and x6 were non-linear in all data sets after IPs were removed. x1 non-linear in C250 |
| **P5:** presentation of functions selected in A5 | 250 (A/B/C) | y, x1, x5, x6 | **Fig 5.** Similar functions estimated for x1, x5 and x6 when IPs removed. Low power for detecting non-linearity in x1 |
| **A6:** MFP models in full data and in data without IPs | 250 (A/B/C) | y, x1-x10 | **Table 3.** Investigate replicability of MFP models in too small datasets. Several deviations from the true model (See Fig 6). For effect of sample size see Tab 4, Tab A5 and Fig 8. |
| **A7a, A7b:** check for IPs in selected MFP model (see A6); L-1 and L-2 | A250 | **y, x1, x3, x5, x6, x10** | **Fig A4.** IPs only identified in x5  **DNS** for L-2 |
| **P6:** x5 function in A7b | A250 | **y, x5** | **Fig A5**. Effects of deleting pair (37, 175) and (151, 175) |
| **A8a, A8b, A9a, A9b** as in A7a, A7b | B250  C250 | y,  B(x1,x5, x6)  C(x5, x6, x10) | B250: **Fig A6**. IPs in x1  C250: **Fig A7**. IPs x10. |
| **P7:** functional forms of variables altered by IPs identified in **A7-A9**; multivariable analysis | 250 (A/B/C) | y, x1, x5, x10 | **Fig 6.** Effects of IPs clearly visible in some variables and in some datasets. |
| **A10a, A10b:** check for IPs in univariable analysis; L-1 and L-2 | A125 | y, x1, x3, x5-x7, x10 | DNS**.** IPs in x1, x6 and x7. |
| **A11a:** check for IPs in multivariable analysis; L-1 | A125 | y, x5, x6 | **DNS**. No IPs found. MFP model included x5, x6 and x8 |
| **A11b:** as in A11a; L-2 | A125 | y, x5, x6 | **DNS**. No IPs found |
| **A12a, A12b:** check for IPs in univariable analysis; L-1 | A500 | y, x1-x10 | **DNS**. (L-1) IPs found in x5, x6 and x7. DNS for (L-2) |
| **A13a, A13b:** check for IPs in multivariable analysis; L-1 and L-2 | A500 | y, x1, x3, x5, x6, x10, x4a, x8, x9b | **DNS**. IPs only in x5. x7 was not selected |
| **A14:** effects of sample size and IPs on selected MFP models | A125  A250  A500 | y, x1-x10 | **Table 4.** MFP models from full data and data without IPs |
| **P8:** functional forms from MFP: Effects of sample size and IPs | A125  A250  A500 | y, x1, x3, x5, x6, x10 | **Fig 7.** x5 and x6 had good agreement with the true function in all data. x1 and x3 not selected in A125. Low power. |
| **A15a, A15b**: as A13a and A13b for other data | B500 | y, x1, x3, x5, x6, x10, x2, x4a, x8 | **DNS**. IPs only in x5. x7 was not selected |
| **A16a, A16b: as A13a** and **A13b** for other data | C500 | y, x1, x3, x5, x6, x10, x4a, x8, x9a | **DNS**. IPs only found in x5. x7 was not selected |
| **A17:** check for IPs in multivariable analysis; L-1 | D1000 | y, x1, x3, x5, x6, x10, x4a, x8 | No IPs at 5% level for FSP. Three IPs only in x5 at 1%. Fig A8 |
| **A18:** MFP models in full data and in data without IPs identified in **A12**, **A15,A16 and A17** | 500 (A/B/C)  D1000 | y, x1, x3, x5, x6, x10, x2, x4a, x8, x9a, x9b | **Table A5.** Good agreement between selected models and the true model. True FP2 for x1 always identified. x3 (true linear) always correct.  In D1000, all relevant variables were selected and all noise variables eliminated |
| **P9:** functional forms from MFP | 500 (A/B/C) | y, x1, x3, x5, x6, x10 | **Fig 8.** Functional forms estimated from data without IPs |
| **P10:** functional forms from MFP | D1000 | y, x5 | **Fig A9.** Functional forms estimated from data with and without IPs and true function |
| **A19:** function Selection Procedure (FSP); Univariable analysis | Bodyfat | Pcfat, all 13 covariates | **Table A6.** Variables abdomen, weight, ankle and hip with non-linear effect. Linear function chosen for another 8 variables. Variable height is non-significant. |
| **A20a, A20b:** check for IPs in univariable analysis; L-1 and L-2 | Bodyfat | All variables | **DNS.** IPs in ankle, biceps, abdomen, hip and weight. No IPs in other variables. Same results for L-1 and L-2. |
| **A21:** MFP models in full data and data without IPs identified in A19 | Bodyfat | All variables | **Table A7.** No non-linear function after deleting IPs |
| **P11**: functional forms; multivariable analysis of full data | Bodyfat | Pcfat, biceps, abdomen, height, wrist | **Fig A8.** Functional forms for continuous variables selected by MFP (0.05, 0.05). |
| **A22a:** check for IPs in selected MFP (0.05, 0.05) model L-1 | Bodyfat | Pcfat, biceps, abdomen, height,wrist | **DNS.** No IPs found. |
| **A22b: as 22a,** (L-2) | Bodyfat | Pcfat, biceps, abdomen, height and wrist | **DNS.** IPs found in biceps. |

D = description; A = analysis; P-presentation; IPs = Influential Points; L-1 = leave-one-out approach; L-2 = leave-two-out approach; NA = not applicable; DNS = Data not shown

3 More Details on Simulated Data

**3.1 contribution of each variable to the model fit**

Table A2 ART data (N = 5,000, *R^2^*=0.49). Contribution of each predictor to the model fit, expressed in terms of the percentage reduction in *R^2^* when regressing the index on all predictors minus the one of interest. The last column shows the variables that was used to generate the outcome variable

| Variable | Model^a^ | R^2^(-xi) | % reduction *R^2^* | True model |
| --- | --- | --- | --- | --- |
| x1 | 0.5, 1 | 0.47 | 4.8 | x |
| x2 | 1 | 0.49 | 0.1 | - |
| x3 | 1 | 0.47 | 5.0 | x |
| x4a | 1 | 0.48 | 3.4 | x |
| X4b | 1 | 0.49 | 0.0 | - |
| x5 | -0.2 | 0.22 | **56.0** | x |
| x6 | 0 | 0.41 | **17.1** | x |
| x7 | 1 | 0.49 | 0.1 | - |
| x8 | 1 | 0.46 | 6.0 | x |
| x9a | 1 | 0.49 | 0.0 | - |
| X9b | 1 | 0.49 | 0.01 | - |
| x10 | 1 | 0.47 | 5.4 | x |

^a^ are FP powers, “x” denotes a signal variable while “-” denotes a noise variable

**3.2 Description of the Data A250**

For the data A250, we describe the univariable distribution and the correlation structure in detail. This will provide good insight into the design of the simulated data.

*Univariable Distributions*

In real data, initial data analysis needs to be carried out before the formal statistical analysis [5] in order to check for data errors and whether some manipulations are needed such as combining one or more categories with very low percentages. However, this part is less relevant in simulated data and in all data sets used, we assumed that this work has been carefully done. Table A3 presents descriptive statistics for sample A250. It can be seen that variables $y, x1$ and $x10$ have skewness and kurtosis near 0 and 3 respectively, hence they are approximately normally distributed. Variable x5, x6 and x7 have high kurtosis an indication of existence of extreme values or outliers that warrant investigation. In addition, there are only 16 observations (6%) in category 3 of x9. In building a multivariable model, it may be worth combining it with one of the other two categories, whichever is more sensible from a subject-matter point of view. This would be an issue handled in initial data analysis but we decided against combining any categories and used two dummy variables because x9 is a nominal variable.

Table A3. Data A250. Descriptive statistics for continuous (top) and categorical (bottom) variables

| Variable | Mean | Median | SD | Min. | Max. | Skewness | Kurtosis |
| --- | --- | --- | --- | --- | --- | --- | --- |
| y | 12.17 | 12.10 | 0.99 | 8.28 | 15.15 | -0.04 | 3.72 |
| x1 | 54.24 | 54.00 | 9.69 | 24.00 | 85.00 | 0.16 | 2.98 |
| x3 | 20.78 | 19.00 | 9.31 | 5 | 81.00 | 1.93 | 10.44 |
| x5 | 6.90 | 3.16 | 16.94 | 0.46 | 234.34 | 10.27 | 132.50 |
| x6 | 147.92 | 80.00 | 184.24 | 0.00 | 1200.00 | 2.44 | 10.44 |
| x7 | 105.96 | 54.50 | 169.85 | 0.00 | 1727.00 | 5.26 | 43.36 |
| x10 | 16.95 | 15.90 | 8.32 | 0.61 | 43.46 | 0.70 | 3.35 |

| Variable | 0 | 1 | 2 | 3 |
| --- | --- | --- | --- | --- |
| x2 | 62 (24.80%) | 188 (75.20%) |  |  |
| x4 |  | 30 (12%) | 164 (65.6%) | 56 (22.4%) |
| x8 | 165 (66%) | 85 (34%) |  |  |
| x9 |  | 176 (70.4%) | 58 (23.2%) | 16 (6.4%) |

*Correlation Structure*

Table A4 provides a summary of Spearman rank correlation coefficients and their differences with Pearson correlation coefficients. Only Spearman correlation coefficients whose absolute values were greater than 0.25 and differences greater than 0.05 were shown. Larger differences between the two types of correlation coefficients may indicate presence of outliers or stronger non-linear relationships. Given that the Spearman correlation is more robust against outliers than the Pearson correlation, the largest difference between y and x5 suggests that variable x5 may contain outliers.

Table A4. Data A250. The entries above and below the main diagonal are Spearman correlation coefficients with absolute values larger than 0.25 and differences between Spearman and Pearson correlation coefficients greater than 0.05 for continuous variables.

|  | y | x_1_ | x_3_ | x_5_ | x_6_ | x_7_ | x_10_ |
| --- | --- | --- | --- | --- | --- | --- | --- |
| y | - | · | · | -0.45 | · | · | · |
| x_1_ | · | - | · | · | · | · | · |
| x_3_ | · | · | - | -0.30 | · | · | · |
| x_5_ | -0.25 | · | -0.15 | - | 0.29 | · | · |
| x_6_ | · | · | · | 0.10 | - | 0.40 | · |
| x_7_ | · | · | · | -0.11 | · | - | · |
| x_10_ | · | · | · | · | · | · | - |

4 Results

## 4.1 Univariable Analysis

***Identification of Influential Points in Data Set B250 and C250***

In B250, three IPs (95, 160, and 223) were only identified in variable x7, with obs. 223 having a substantial influence on both the selection of variable x7 and its functional forms. For instance, the removal of this observation made variable x7 be included in the model (Table 2) and the test of FP2 vs. linear significant, implying a non-linear function.

Several IPs were observed in data set C250 (Figure A1). The estimated FP2 (3, 3) function for x1 (Table 2) was purely driven by an influential obs.213, whose removal led to a linear function. Further investigation of six IPs reported in variable x6 using the leave-two-out approach revealed that out of six observations, only obs. 52 was highly influential as expected since the other five observations were borderline significant. Besides obs. 52, other IPs were identified, but the residual plots (Figure A2, lower panel) suggested that a non-linear function was a better fit than a linear function obtained by deleting all IPs; hence only obs. 52 was considered an influential point in variable x6. Furthermore, in C250, observations 133 and 209 were considered IPs in variable x5 in the leave-two-out approach. Removal of these two observations led to an FP2 (0.5, 0.5) function. An inspection of the residual plot (Figure A2, upper panel) supported an FP2 rather than an FP1 (0) function estimated from the complete data, thus observations 133 and 209 were considered IPs. Although several IPs for variable x7 were reported in single case deletion, all of them were inconsequential based on leave-two-out except case 104, which had the largest deviance difference when individually deleted. However, its deletion produced a complex FP2 (2,-0.5) function that was quite similar to the estimated function from the complete data except at the beginning where a hook was visible (Figure A3). Sauerbrei and Royston [6] experienced a similar outcome and suggested the use of pre-transformation of the variable before fitting an FP model. Here, we retained this observation and allowed the maximum permitted function to be an FP1, which produced an FP1(0) function as shown in Figure A3.


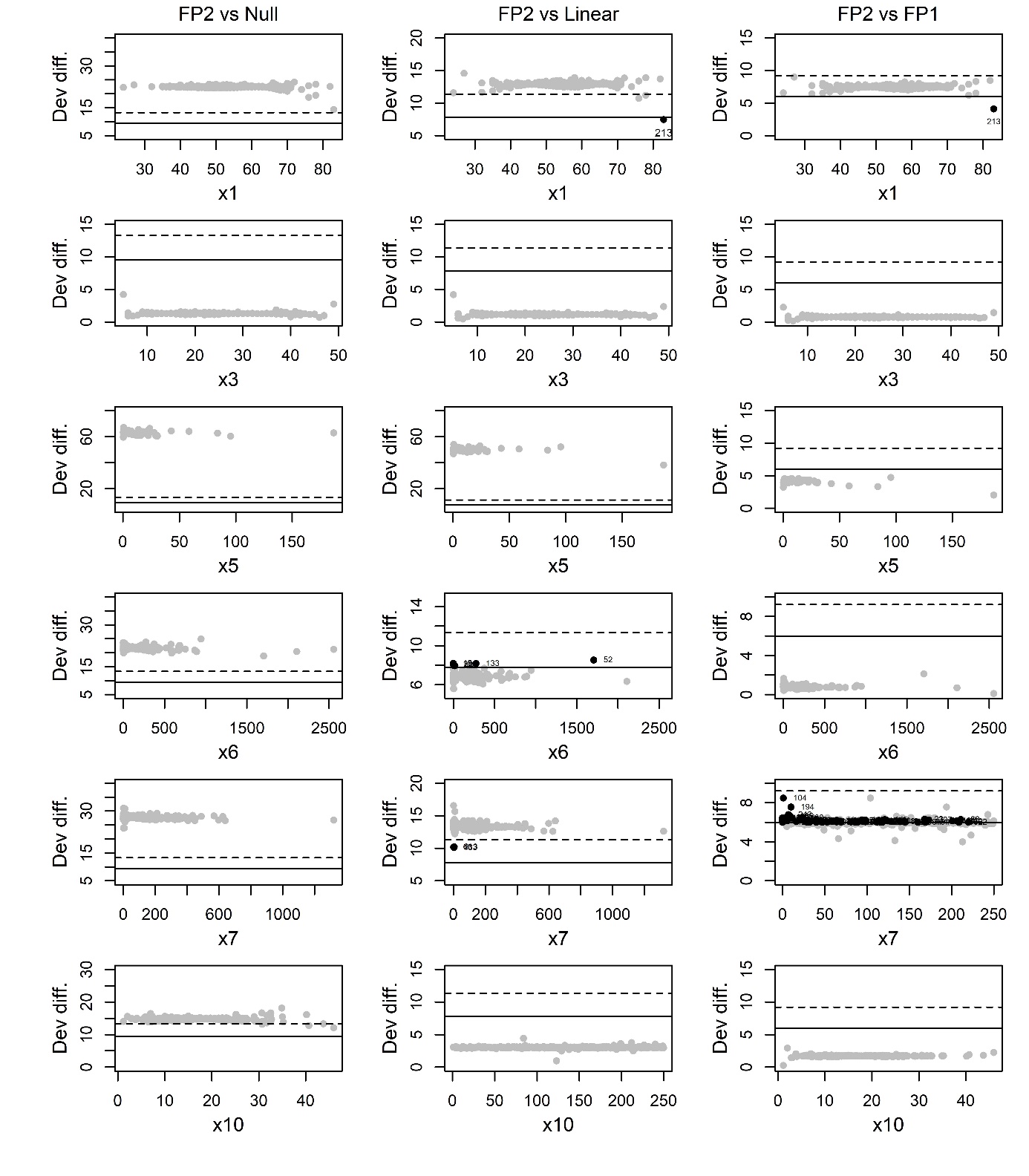


Figure A1 Data C250. Identification of influential points in univariable analysis using leave-one-out approach


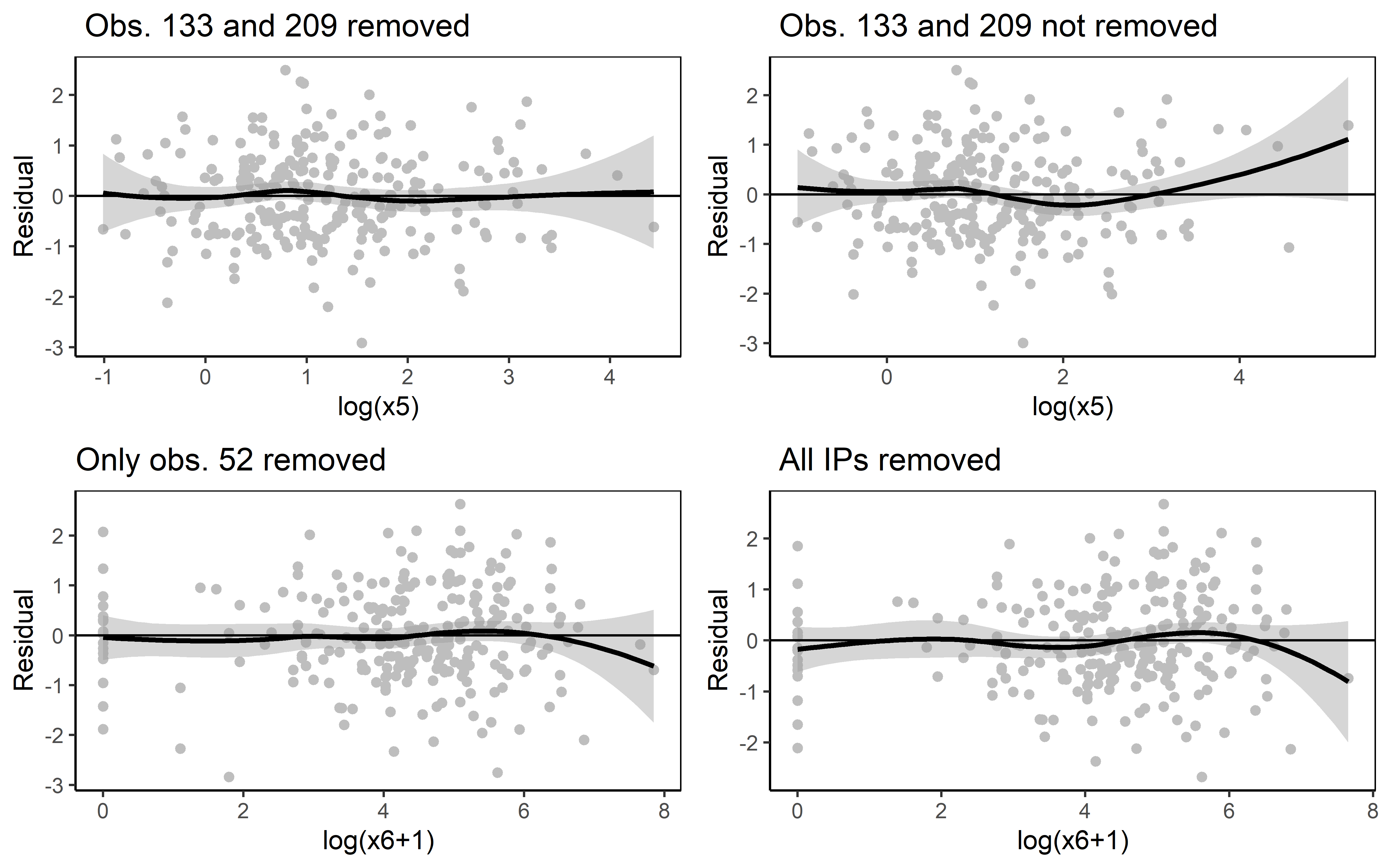


Figure A2. Data C250, univariable analysis. Smoothed residuals with 95% pointwise confidence intervals for variable x5 and x6 before and after removal of IPs.

***
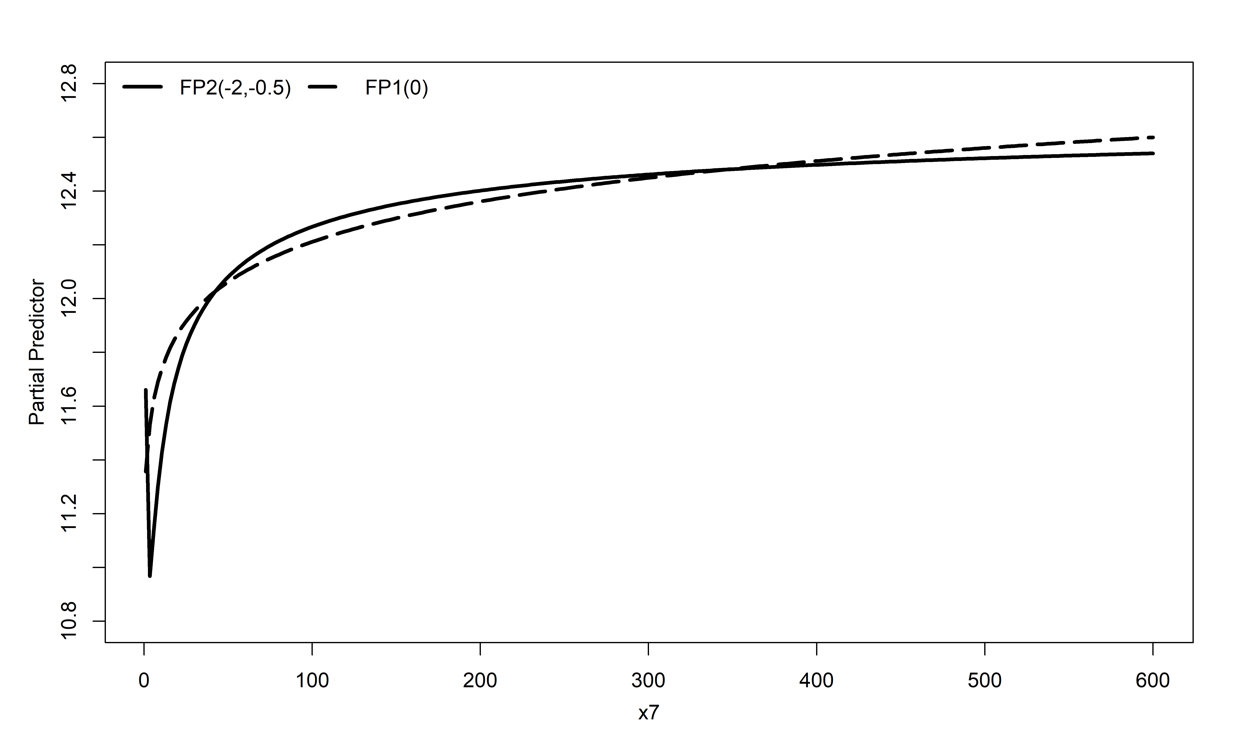
***

**Figure A3** Data C250. Functional form of variable x7 in full data (dashed line) and without observation 104 (solid line). Truncated at 600.

**4.2 *Results of Multivariable Analysis***

***Identification of Influential Points in Data A250***


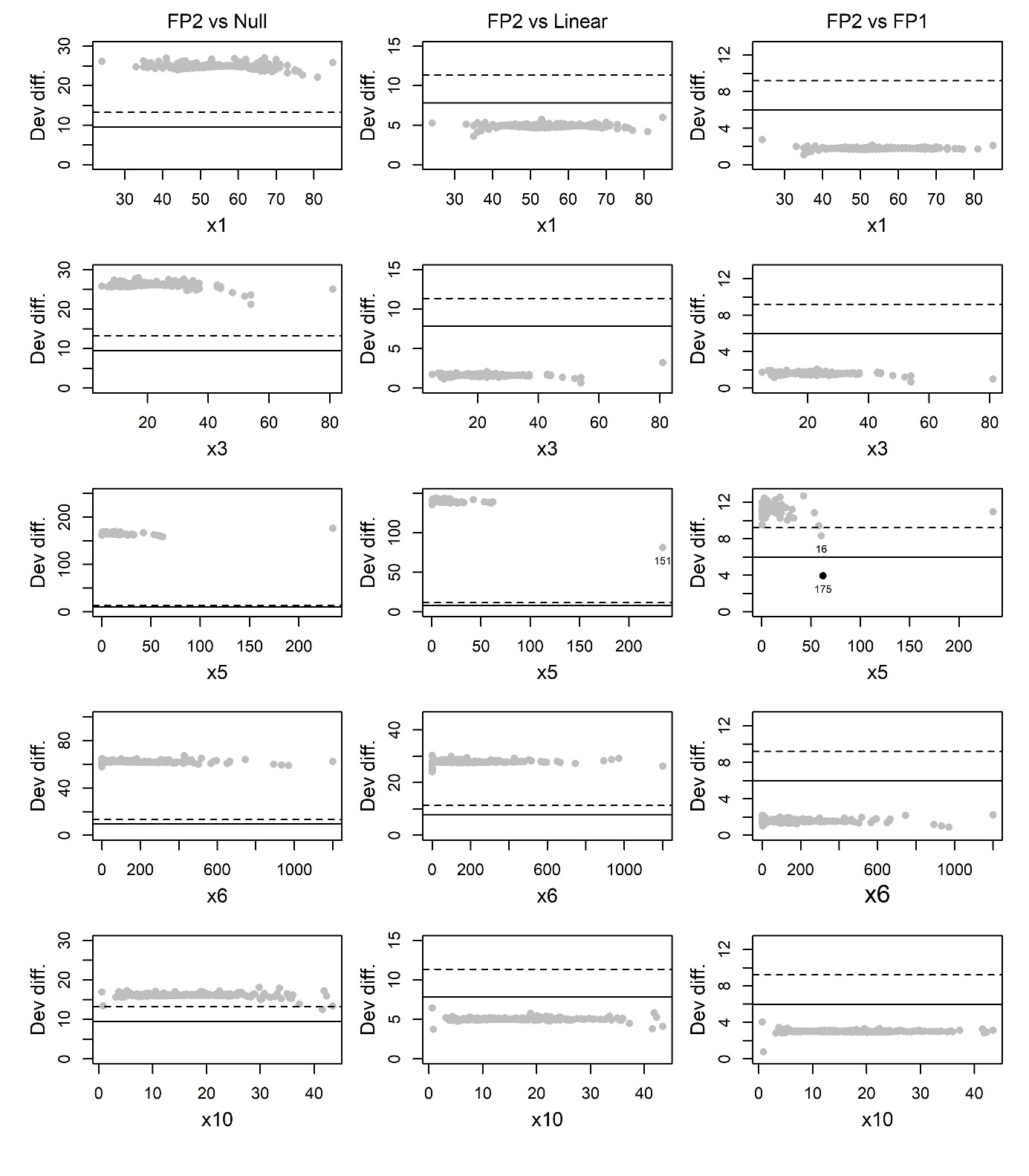


Figure A4 Data A250. Identification of influential points using L-1 approach in the selected MFP model (see Table 3, all data)


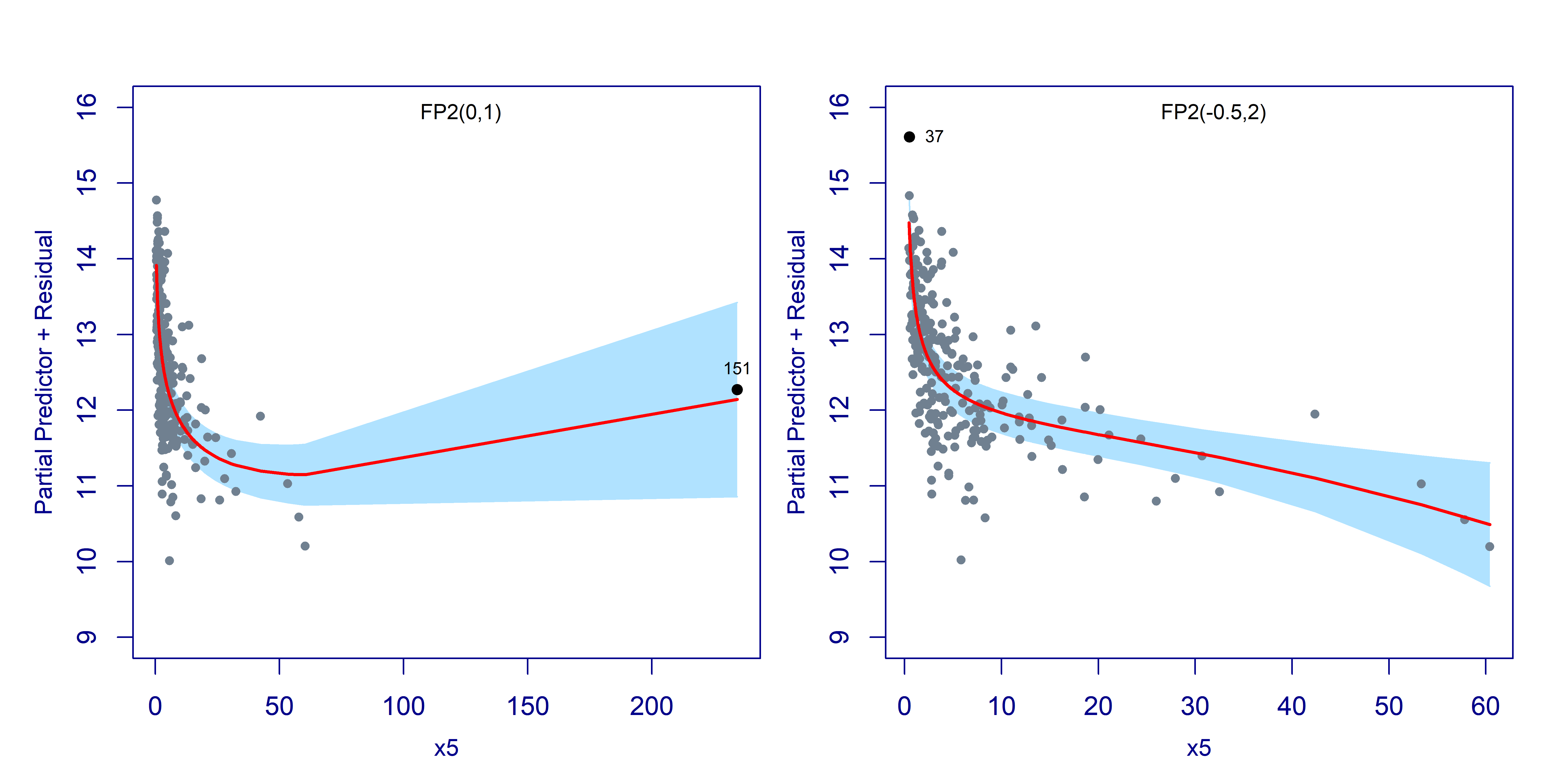


Figure A5 Data A250. Identification of influential points in multivariable analysis using leave-two-out approach. Left panel: functional form for x5 when the pair (37, 175) was removed. Right panel: functional form for x5 when pair (151, 175) was removed.

***Identification of Influential Points in Data B250 and C250***

In data B250, four IPs were solely identified in variable x1 using the leave-one-out approach. The deletion of any of these observations made the test of FP2 vs. FP1 non-significant (Figure A6). Interestingly, the leave-two-out approach disclosed that the test of FP2 vs. FP1 was not necessary since it was only driven by obs. 6. The pair (6, 221) made the test of FP2 vs. linear significant. When these two observations were removed, a linear function was sufficient to describe variable x1. As a result, observations 6 and 221 were considered influential points. Observation 98 was not considered influential, yet it had an extremely large value of variable x5. For this reason, the observation was removed before the functional plots were constructed and similar results were obtained. In total, 3 observations (6, 98, and 221) were completely removed from the analysis, as shown in Table 3. From Table 3, two situations were observed in the comparison of “all”, “IPAm” and “true” models. First, a linear function was estimated for variable x1 when the three influential observations were deleted rather than an FP2 (-1, 3) function obtained from complete data. It was obvious that a linear function was not a good approximation of the true FP2 (0.5, 1) function (Figure 6), which can be attributed to the low power of detecting non-linearity. Secondly, when IPs were removed, variable x8 was included in the model, which was initially excluded.


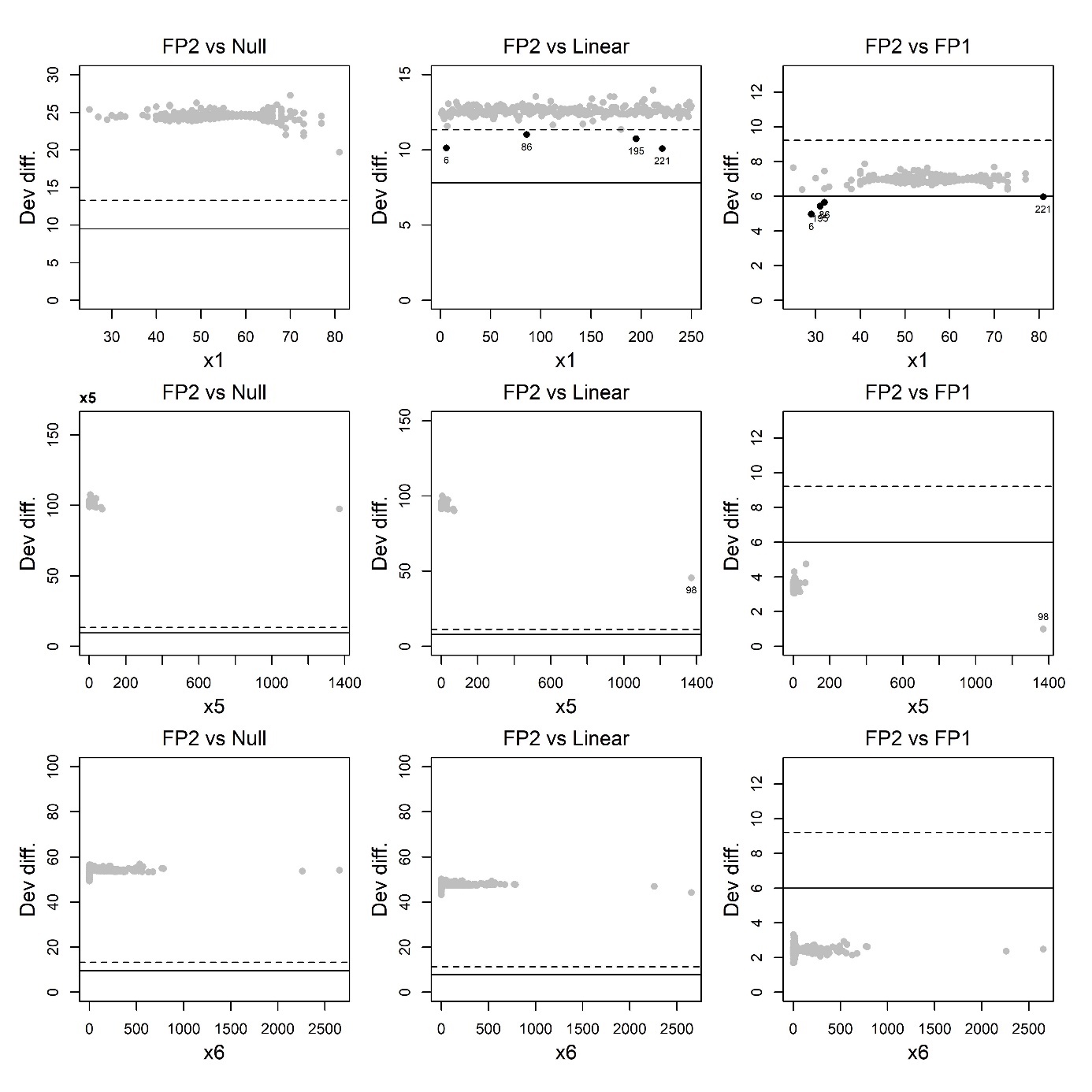


Figure A6 data B250. Identification of influential points in the selected MFP model (see Table 3). Multivariable analysis using L-1 approach

In data C250, the leave-one-out approach identified several IPs in variable x10 that made the test of FP2 vs. linear non-significant, though most were at borderline except six observations, out of which obs. 138 had the largest influence (Figure A7). Moreover, the leave-two-out approach identified six observations (82, 123, 129, 138, 194, and 214) with substantial influence on variable x10, which also correspond to the first six observations with large deviance differences when individually deleted. Only observation 123 was influential in both univariable and multivariable analysis. From Table 3, the two models (“all” and “IPAm”) are similar except that variable x10 was excluded when IPs were deleted.


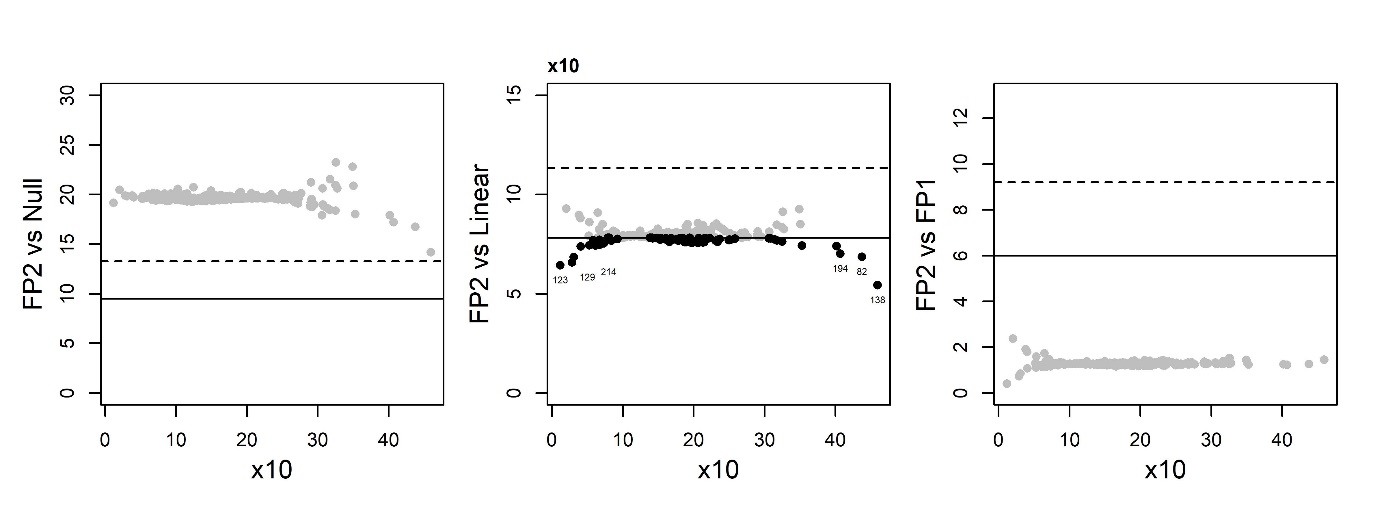


Figure A7 data C250. Identification of influential points of x10 in the selected MFP model using L-1approach

**4.3 Identifiability of the MFP models in relatively large datasets**

Table A5 Data A500, B500, C500 and D1000. Multivariable analysis for relatively large datasets. See Figure 8, A8 and A9 for the related functions.

|  | **A500(1)** | | **B500(1)** | | **C500(1)** | | **D1000(3)** | |  |
| --- | --- | --- | --- | --- | --- | --- | --- | --- | --- |
| **Variables** | **All** | **IPAm** | **All** | **IPAm** | **All** | **IPAm** | **All** | **IPAm** | **True model** |
| x1 | 0.5, 1 | = | -0.5, 3 | 0, 3 | 2, 2 | = | 1, 1 | 1, 1 | 0.5, 1 |
| x3 | 1 | = | 1 | = | 1 | = | 1 | = | 1 |
| x5 | 0, 3 | 0 | -0.5, -0.5 | 0 | 0, 3 | 0 | 0, 0.5 | 0 | -0.2 |
| x6 | 0 | = | 0 | = | 0 | = | 0 | = | 0 |
| x7 | out | = | out | = | out | = | out | = | Out |
| x10 | 1 | = | 1 | = | 3 | = | 1 | = | 1 |
| x9a | out | = | in | = | out | = | out | = | Out |
| x9b | in | out | out | = | out | = | out | = | Out |
| x2 | out | = | out | = | in | = | out | = | Out |
| x4a | in | = | in | = | in | = | 1 | = | In |
| x4b | out | in | out | = | out | = | out | = | Out |
| x8 | in | = | in | = | in | = | in | = | In |


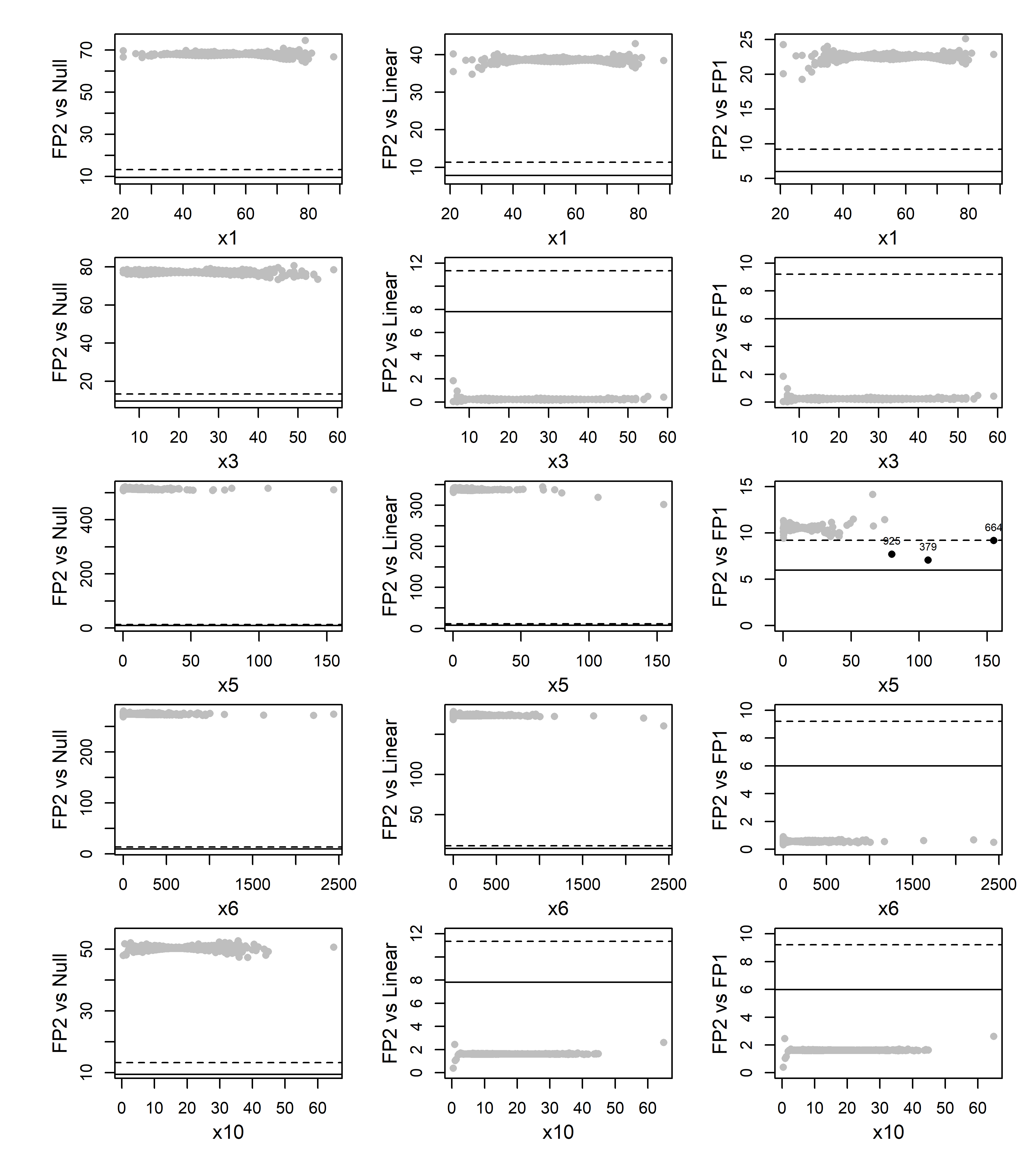


Figure A8. Data D1000. Identification of influential points in the selected MFP model (see Table A5). Multivariable analysis using L-1 approach. No IPs identified at 5% level, but 3 IPS identified at 1% in variable x5.


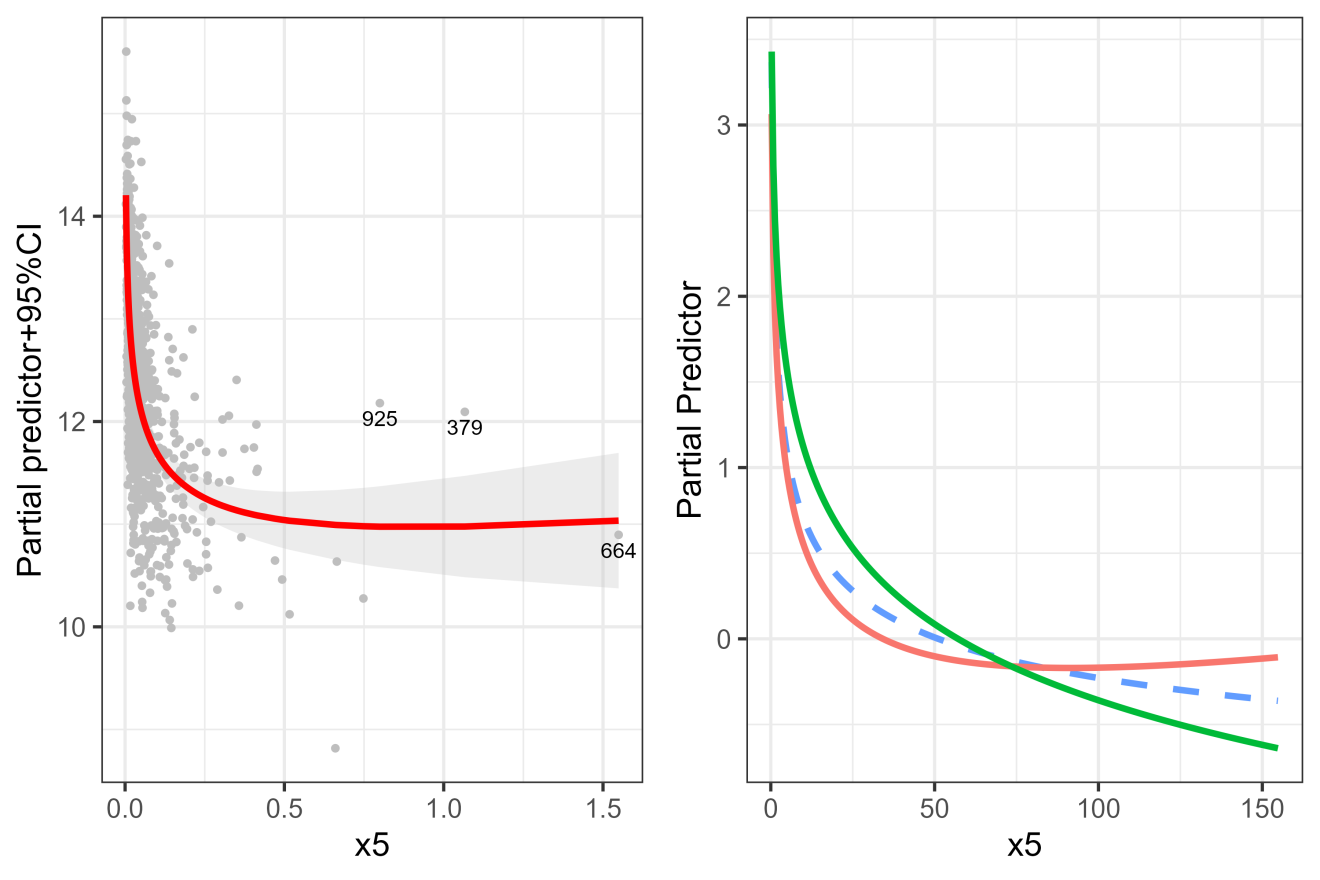


Figure A9 Data D1000. Identification of influential points in multivariable analysis using L-1. Left panel: functional form for x5 in full data. Right panel: functional form for x5 before (red solid line) and after (blue dashed line) removal of observations 379, 664 and 925. The green solid line is the true function.

5 Case study-body fat data

*5.1 Univariable Analyses*

Table A6 shows the results of the function selection procedure. All variables except height (p=0.994) were considered important predictors of body fat at the 0.05 significance level. Four variables (abdomen, weight, ankle, and hip) were found to be non-linear, as shown in the test of FP2 vs. linear.

Leave-one-out approach identified four IPs (31, 39, 182, and 192), whereas leave-two-out approach identified three IPs (31, 39, and 54). Observations 182 and 192 that were influential in the leave-one-out approach were not influential in the leave-two-out approach probably because they were borderline significant. Removal of obs. 39 or any pair with this observation rendered the test of FP2 vs. linear non-significant in univariable models of the abdomen, hips, and weight. Removal of obs.31 in the ankle rendered the test of FP2 vs. linear non-significant. This implies that in univariable analysis, the linearity assumption sufficed for these variables.

Interesting results were found in a univariable model of biceps; deleting any pair with obs.39 produced a non-linear function except when 11 pairs were deleted. Out of these 11 pairs, a pair (39, 54) decreased the deviance difference considerably. Therefore, instead of deleting all the observations that comprise 11 pairs, which perhaps might lead to a loss of power, only pair (39, 54) was deleted. Hence, observations 39 and 54 were considered IPs, their deletions produced a linear function for the biceps (Table A6). Once all IPs were deleted (obs. 31, 39, and 54), it turned out that no covariate was non-linearly related to the outcome variable, as can be seen in Table A6 (last column).

**Table A6**: Data body fat, univariable analysis. P-values for different model comparison are displayed in column 2-4. The last two columns show the FP powers or exclusion of a variable in the complete data set and after deleting influential points respectively.

| Variable | FP2 vs. Null | FP2 vs. Linear | FP2 vs. FP1 | Univariable selection | Univariable selection after 3 IPs deleted* |
| --- | --- | --- | --- | --- | --- |
| Biceps | 0.000 | 0.229 | 0.903 | 1 | 1 (39, 54) |
| Abdomen | 0.000 | **0.000** | 0.036 | **3,3** | 1(39) |
| Height | **0.994** | 0.995 | 0.967 | Out | Out |
| Wrist | 0.000 | 0.862 | 0.906 | 1 | 1 |
| Weight | 0.000 | **0.019** | 0.786 | **-0.5** | 1(39) |
| Neck | 0.000 | 0.811 | 0.644 | 1 | 1 |
| Thigh | 0.000 | 0.079 | 0.932 | 1 | 1 |
| Ankle | 0.000 | **0.044** | 0.165 | **-2** | 1(31) |
| Forearm | 0.000 | 0.390 | 0.228 | 1 | 1 |
| Age | 0.000 | 0.808 | 0.673 | 1 | 1 |
| Chest | 0.00 | 0.518 | 0.819 | 1 | 1 |
| Hip | 0.000 | **0.008** | 0.575 | **-2** | 1(39) |
| Knee | 0.000 | 0.864 | 0.971 | 1 | 1 |

*The number enclosed in brackets are the identified influential points

5.2 Multivariable Analyses

The MFP (0.05, 0.05) was applied to complete body fat data, and four variables were selected: biceps, abdomen, height, and wrist. These variables explained about 75% of the total variation as shown in Table A7. Analysis of the contribution of each variable to the model fit showed that the abdomen was the most important predictor since its omission led to a reduction of R^2^ by about 60%. Similarly, its contribution to the “full” model obtained with MFP (1, 0.05) was also large (about 17%). Biceps was the only non-linear variable with an FP2 (3, 3) function. An inspection of its functional form (Figure A8) showed that an obs. 39 with the largest value of biceps caused an FP2 function. Hence, there is a need to check for influential points.

In the univariable approach, three IPs (31, 39, and 54) were identified. After removing them, a different MFP model was selected (see Table A7, column IPBFu) where biceps and height were excluded, while weight and thigh were included with linear and FP2 (-2, -2) functions, respectively.

In this subsection, we present the results of the diagnostic check of IPs in the selected MFP model with four variables: biceps (3,3), abdomen (1), height (1), and wrist (1). The leave-one-out approach did not identify any influential points, while the leave-two-out approach identified IPs in the biceps. Deleting any pair with observation 39 made the test of FP2 vs. null borderline significant except that it became non-significant when 17 pairs were deleted. Moreover, deleting two pairs (39, 172) and (39, 180) rendered the test of FP2 vs. linear non-significant. This implies that, besides obs.39, other influential points existed in the data. Among the 17 pairs, deleting a pair (39, 172) decreased the deviance difference the most (in FP2 vs. null), followed by (39, 180), while others were close to the borderline. To avoid loss of information resulting from deleting all 17 pairs, we only considered observations 39, 172, and 180 as influential points in the multivariable model. The results are summarized in Table A7 (column labelled IPBFm). The removal of these three IPs led to the exclusion of biceps and height, while weight was included in the model. Furthermore, all the selected continuous variables were described by a linear function, so non-linearity was driven by IPs.

**Table A7** Data body fat. Selected models from a MFP analysis with all data represented by MFP(0.05, 0.05) and after removal of IPs identified from univariable (IPBFu (k)) and multivariable (IPBFm(l)) diagnostic analyses where k and l represent the number of IPs identified in the corresponding analysis. MFP (1, 0.05) – no elimination of variables, FSP with significance level 0.05.

| Variable | MFP (0.05, 0.05) | |  | MFP (1, 0.05) | |  |  | IPBFu (3)* | IPBFm (3)* |
| --- | --- | --- | --- | --- | --- | --- | --- | --- | --- |
|  | Model^a^ | R^2^ _red_ |  | Model^a^ | R^2^_Red_ |  |  | Model^a^ | Model^a^ |
| Biceps | 3, 3 | 4.32 |  | 1 | 0.09 |  |  | out | = |
| Abdomen | 1 | 59.82 |  | 0 | 16.79 |  |  | 1 | = |
| Height | 1 | 1.69 |  | 1 | 0.37 |  |  | out | = |
| Wrist | 1 | 2.63 |  | 1 | 1.52 |  |  | 1 | = |
| Weight | Out | - |  | 1 | 0.01 |  |  | 1 | = |
| Neck | Out | - |  | 1 | 0.63 |  |  | out | = |
| Thigh | Out | - |  | 1 | 0.12 |  |  | -2, -2 | out |
| Ankle | Out | - |  | 1 | 0.07 |  |  | out | = |
| Forearm | Out | - |  | 1 | 0.41 |  |  | out | = |
| Age | Out | - |  | 1 | 0.54 |  |  | out | = |
| Chest | Out | - |  | 1 | 0.08 |  |  | out | = |
| Hip | Out | - |  | 1 | 0.12 |  |  | out | = |
| Knee | Out | - |  | 1 | 0.01 |  |  | out | = |
| R^2^ | 0.748 |  |  | 0.757 |  |  |  | 0.745 | 0.737 |

^a^ Numbers are FP powers; ^b^ R^2^_Red,_ denotes percentage reduction in R^2^ by eliminating a variable from the selected MFP model (see also Table A2). IPs 31, 39 and 54 were identified in IPBFu while obs.39, 172 and 180 were identified in IPBFm.


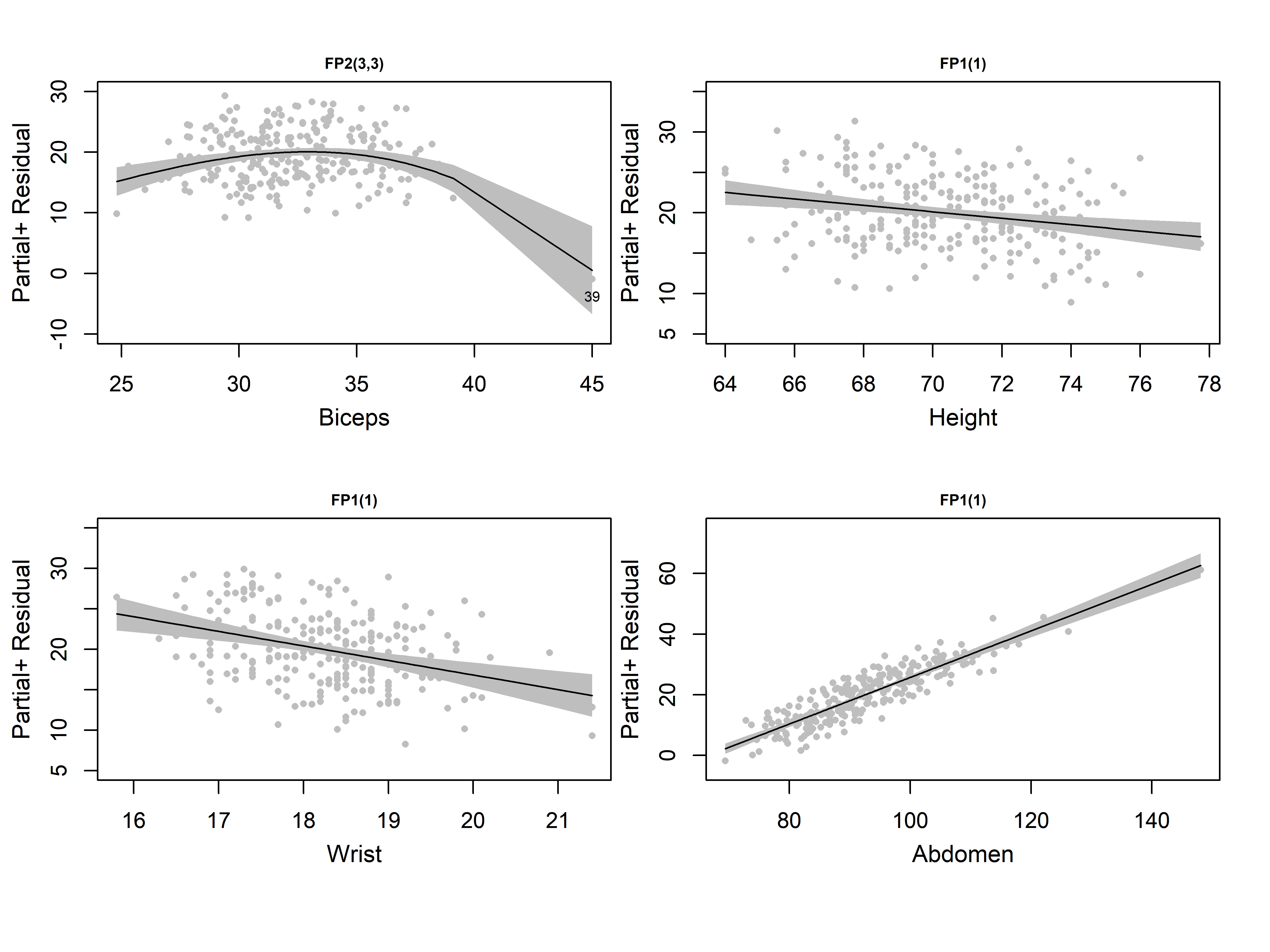


**Figure A10** Data body fat. Multivariable analysis of complete data. Functional forms for continuous predictors in MFP (0.05, 0.05) model. Deleting 3 IPs biceps is no longer significant.

**References**

1. Morris TP, White IR, Crowther MJ. Using simulation studies to evaluate statistical methods. Statistics in medicine. 2019;38(11):2074-102.
2. Altman D.G., McShane L., Sauerbrei W., Taube S.E. Reporting recommendations for tumor marker prognostic studies (REMARK): explanation and elaboration. PLoS Med. 2012;9(5):e1001216.
3. Winzer K.J., Buchholz A., Schumacher M., Sauerbrei W. Improving the prognostic ability through better use of standard clinical data - the Nottingham Prognostic Index as an example. PLoS ONE. 2016;11(3):e0149977.
4. Johnson RW. Fitting percentage of body fat to simple body measurements. Journal of Statistics Education. 1996;4(1).
5. Huebner M, le Cessie S, Schmidt C, Vach W on behalf of the Topic Group “Initial Data Analysis” of the STRATOS Initiative. A Contemporary Conceptual Framework for Initial Data Analysis. Observational Studies. 2018;(4):171-192.
6. Sauerbrei, W. and Royston, P. Building multivariable prognostic and diagnostic models: transformation of the predictors by using fractional polynomials. Journal of the Royal Statistical Society. Series A (Statistics in Society). 1999;162:71–94.
